# Multitier mechanics control stromal adaptations in the swelling lymph node

**DOI:** 10.1038/s41590-022-01257-4

**Published:** 2022-07-11

**Authors:** Frank P. Assen, Jun Abe, Miroslav Hons, Robert Hauschild, Shayan Shamipour, Walter A. Kaufmann, Tommaso Costanzo, Gabriel Krens, Markus Brown, Burkhard Ludewig, Simon Hippenmeyer, Carl-Philipp Heisenberg, Wolfgang Weninger, Edouard Hannezo, Sanjiv A. Luther, Jens V. Stein, Michael Sixt

**Affiliations:** 1 Institute of Science and Technology Austria (ISTA), Klosterneuburg, Austria; 2 Department of Dermatology, Medical University Vienna, Vienna, Austria; 3 Department of Oncology, Microbiology and Immunology, University of Fribourg, Fribourg, Switzerland; 4 BIOCEV, First Faculty of Medicine, Charles University, Vestec, Czech Republic; 5 Institute of Immunobiology, Kantonsspital St Gallen, St Gallen, Switzerland; 6 Department of Immunobiology, University of Lausanne, Epalinges, Switzerland

**Keywords:** Lymph node, Antigen presentation

## Abstract

Lymph nodes (LNs) comprise two main structural elements: fibroblastic reticular cells that form dedicated niches for immune cell interaction and capsular fibroblasts that build a shell around the organ. Immunological challenge causes LNs to increase more than tenfold in size within a few days. Here, we characterized the biomechanics of LN swelling on the cellular and organ scale. We identified lymphocyte trapping by influx and proliferation as drivers of an outward pressure force, causing fibroblastic reticular cells of the T-zone (TRCs) and their associated conduits to stretch. After an initial phase of relaxation, TRCs sensed the resulting strain through cell matrix adhesions, which coordinated local growth and remodeling of the stromal network. While the expanded TRC network readopted its typical configuration, a massive fibrotic reaction of the organ capsule set in and countered further organ expansion. Thus, different fibroblast populations mechanically control LN swelling in a multitier fashion.

## Abstract (web summary)

Sixt and colleagues show that different fibroblast populations in the lymph node mechanically control its swelling in a multitier fashion.

## Main

Opposed to other organs, the LN parenchyma contains few resident cells, while the bulk of lymphocytes is in constant transit and millions of them pass through the organ every day^1–3^. Despite this dynamic cellular exchange, homeostatic LN size remains relatively stable. Known modulators of homeostatic LN cellularity (for example, during circadian rhythms^4,5^) are mainly chemoattractants and adhesion molecules^2,6–9^, which serve as entry and exit signals for lymphocytes, as well as survival factors^10–13^ and adrenergic signals^14,15^. The main stromal cells of LNs are fibroblastic reticular cells (FRCs), a heterogeneous group of cells that form the non-haematopoietic backbone of the organ^16^. Like glial cells of the nervous system, FRCs were long considered passive structural elements. Only in the last two decades has it been revealed that the stromal compartment decisively orchestrates immune cell encounters by providing trophic and tactic cues and that, in turn, FRCs dynamically respond to signals provided by the immune cells^17,18^. TRCs are the largest FRC subset and deposit bundled fibers of extracellular matrix (ECM) that assemble an intricate three-dimensional (3D) network termed conduits. TRCs enwrap these ECM conduits and form an interface with the immune cells, while conduits associated with other FRCs are scarce^19,20^.

Upon immunological challenge, reactive LNs swell rapidly by recruiting large numbers of naïve lymphocytes via high endothelial venules (HEVs), while lymphocyte egress via efferent lymphatics is initially blocked^21,22^. LNs can swell up to tenfold in size in the order of days, imposing a structural problem on the stromal network that has to cope with this volumetric challenge. TRCs are able to relax and expand upon interaction with activated DCs^23,24^, potentially creating additional space during the swelling phase. In addition, TRCs increase in number and various redundant mechanisms that drive this expansion in the early and late phase of LN swelling have been described^21,23–25^. The ratio of TRC-to-lymphocyte numbers remains fairly constant in the swelling LN, and trapping of naïve lymphocytes in the absence of inflammatory stimuli has been demonstrated as a sufficient stimulus for the TRC network to grow^21^. How network expansion is coordinated to prevent undergrowth or overgrowth of the TRC network is unknown, and, although mechanical forces are obvious feedback parameters, these aspects of LN swelling have not been measured to date.

Here we investigated the cellular and mechanical changes accompanying LN swelling and show that mechanical load on the conduit network and subsequent TRC mechanosensing are central to expansion of the TRC network and LN growth.

## Results

### The reactive lymph node resists swelling

**Fig. 1:**
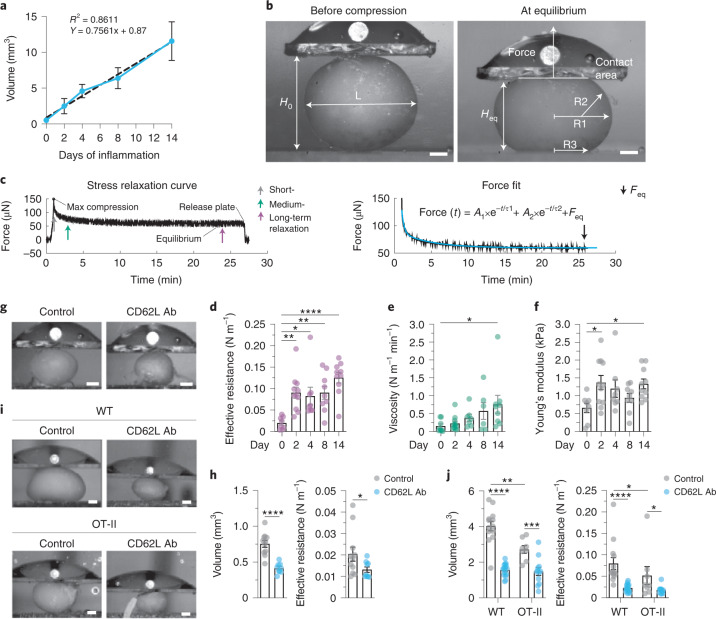
The reactive lymph node resists swelling. **a**, Volumes of swelling LNs calculated from 2D side views over the course of 2 weeks after immunization (*n* = 46). Means are connected (blue line) and a linear regression line (dashed) has been fitted to the data. **b**, Measured geometrical parameters annotated on 2D side images during a measurement (25% strain). Force is measured on the top plate. Scale bar, 300 µm. *H*_0_, LN height before compression; L, LN length before compression; *H*_eq_, LN height at equilibrium. R1, R2 and R3 indicate measured radii. **c**, Stress relaxation curve from the measured force over time (left) and the corresponding force fit (right). Colored arrows indicate short-, medium- and long-term relaxations. Force is fitted with a double exponential equation (blue line). The arrow (black) indicates force at equilibrium (*F*_eq_). **d**–**f**, Quantification of the effective resistance (**d**; *n* = 8, 11, 8, 9 and 10), viscosity (**e**; *n* = 8, 11, 7, 6 and 10) and Young’s modulus (**f**; *n* = 8, 11, 8, 9 and 10). **g**–**j**, Stress relaxation measurements in LNs of wild-type (WT) mice during homeostasis (day 0; **g** and **h**) and in LNs of wild-type or OT-II mice during inflammation (day 4; **i** and **j**) following treatment with PBS or CD62L antibody intravenously injected 24 h before measurements at day 0 or injected at immunization for measurements at day 4. **g**,**i**, Representative side views of explanted and measured LNs. Scale bars, 300 µm (**g**) and 400 µm (**i**). **h**, Quantification of LN volume (left, *n* = 11 and 9) and quantification of effective resistance (right, *n* = 11 and 9). **j**, Quantification of LN volume (*n* = 13, 16, 8 and 12) and effective resistance (*n* = 13, 16, 8 and 11). Data from **a**, **d**–**f**, **h** and **j** are shown as the mean ± s.e.m. and individual datapoints represent independent measurements of single popliteal LNs. Statistical analysis was performed using Kruskal-Wallis test (**d**–**f**), unpaired two-tailed *t*-test (**h**; left), two-tailed Mann-Whitney test (**j**; left; *y* = (*y*^0.8–1^)/0.8 transformed) and two-way analysis of variance (ANOVA; **h**, right, **j**, right; *y* = ln(*y*) transformed). All experiments were repeated independently (≥5 mice and ≥3 experiments). For statistical details, see Supplementary Table 1. *P < 0.05, **P < 0.01, ***P < 0.001, ****P < 0.0001. Source data

**Extended Data Fig. 1:**
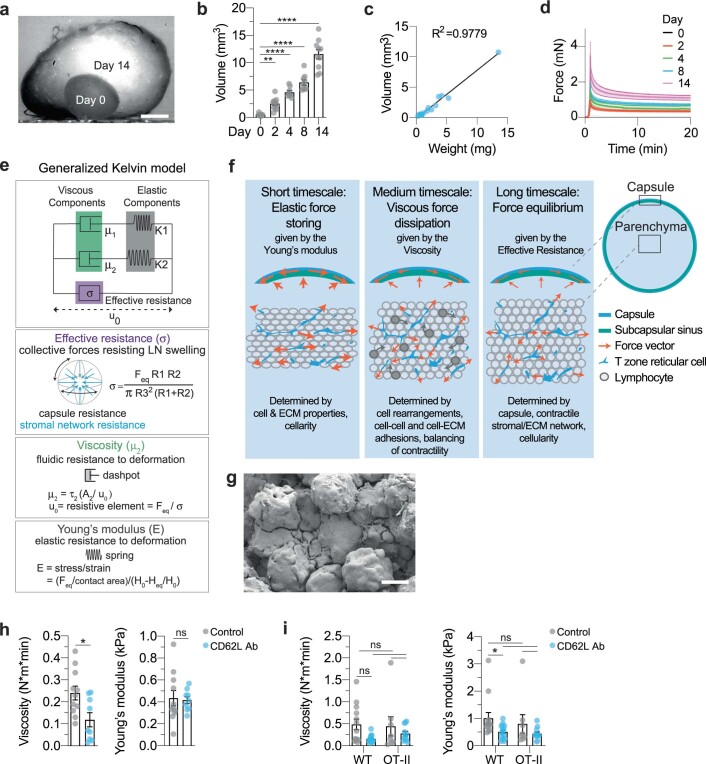
The reactive lymph node resists swelling. (**a**) Side view focus-stack of a homeostatic (day 0) and inflamed (day 14) popliteal lymph node (LN) from wild-type (WT) mice. Scale bar = 500 µm. (**b**) Quantification of LN volumes in homeostasis (day 0) and inflammation (day 2, 4, 8 and 14) from wild-type mice (n = 8, 11, 8, 9, 10). (**c**) Relation between volumes and corresponding measured weights of LNs as in **a**. A regression line is fitted. (**d**) Stress relaxation curves of homeostatic (day 0) and inflamed (day 2, 4, 8 and 14) LNs (n = 6, 6, 6, 6, 6). (**e**) Schematic representation of the generalized Kelvin model used to derive the effective resistance, viscosity and Young’s modulus from stress relaxation experiments on explanted popliteal LNs from wild-type mice in homeostasis (day 0) and inflammation (day 2, 4, 8 and 14). Adapted from *Forgacs et al*.^26^ (**f**) Schematic illustration and description of the short-, medium- and long-term relaxation events in a LN during a stress relaxation experiment. (**g**) Scanning electron microscopy image of packed lymphocytes in the homeostatic (day 0) LN paracortex of a wild-type mouse. Scale bar = 2 µm. (**h**, **i**) Quantification of (**h**; left, **i**; left) viscosity (n = 11, 9 and n = 11, 9) and (**h**; right, **i**; right) Young’s modulus (n = 13, 16, 8, 11) and (n = 13, 16, 8, 11), respectively, of stress relaxation measurements in (**h**) LNs of wild-type (WT) mice in homeostasis (day 0) and in (**i**) LNs of wild-type or OT-II mice during inflammation (day 4) following treatment with PBS or CD62L Ab (i.v. injected 24 h before measurements for homeostasis or at immunization for inflammation). Data from **b**, **d**, **h**, **i** shown as mean ± s.e.m. Datapoints in **b**, **d**, **h**, **i** represent independently measured LNs. Statistical analysis was performed using one-way ANOVA (**b**), unpaired two-tailed t test (**h**) and two-way ANOVA (**i**; y=Ln(y) transformed). All experiments were repeated independently (≥5 mice and ≥3 experiments). For statistical details see Supplementary Information, table 1. NS, not significant. *P < 0.05, **P < 0.01, ****P < 0.0001. Source data

To understand the global mechanical behavior of LNs while expanding, we quantitatively characterized bulk tissue properties of the reactive LN. Upon immunization of wild-type mice with keyhole limpet hemocyanin in complete Freund’s adjuvant (KLH/CFA), we observed a more than tenfold increase in volume of draining LNs at day 14 after immunization, when the organ reached its maximum size. LN volume was calculated from two-dimensional (2D) side view images and showed a volumetric increase of 0.75 mm^3^ per day and a tripled volume by day 2 of inflammation (Fig. 1a and Extended Data Fig. 1a–c). We measured tissue mechanics by compressing explanted popliteal LNs between two parallel plates at 75% of their original height (25% strain), while the resisting force exerted by the LN on the top plate was measured over a period of 20–60 min (Fig. 1b). During this time, the LN underwent a viscoelastic relaxation behavior and reached a new force equilibrium, which is described by the stress relaxation curve (Fig. 1c and Extended Data Fig. 1d). Together with the geometrical parameters of LNs measured before compression and at the new force equilibrium, the effective resistance (*σ*, surface tension), the viscosity (*µ*_2_, fluidic resistance to deformation by an applied force) and the Young’s modulus (*E*, elastic resistance to deformation by an applied force) of the tissue were derived by modeling the parameters to a generalized Kelvin model (Extended Data Fig. 1e,f)^26^. At equilibrium, the LN resisted the external force exerted by the plate, which, together with LN geometry, sets the effective resistance (given in Newton per meter; Fig. 1c). This parameter describes the collective forces resisting organ expansion and is a measure of how much force is necessary to drive organ swelling by a certain length scale. During expansion (day 0 to day 14), we observed a ~fourfold increase of effective resistance and values remained elevated until the endpoint at day 14 (Fig. 1d). Viscosity only increased in the last phase of swelling, while elasticity was selectively increased from the homeostatic condition at day 2 and day 14 of inflammation (Fig. 1e,f). These data demonstrate that tissue properties of LNs show adaptive dynamics upon swelling and suggest that the mechanical features of the organ resist the forces driving expansion.

### Lymphocyte numbers drive lymph node swelling

We next asked what are the internal forces driving organ expansion. Increased entry, blocked exit and proliferation of lymphocytes are the main factors that increase cellularity within the densely packed node (Extended Data Fig. 1g)^27,28^. We first perturbed lymphocyte entry through the HEVs under homeostatic conditions using an l-selectin antagonizing antibody (CD62L)^29^. At 24 h after CD62L antibody administration, LNs were used for parallel-plate compression experiments. Blocking of lymphocyte entry significantly reduced LN volume, effective resistance and viscosity, while the Young’s modulus remained unchanged (Fig. 1g,h and Extended Data Fig. 1h), suggesting that lymphocyte influx represented an internal force that balanced the effective resistance of the homeostatic LN.

Next, we asked how lymphocyte cellularity affected LN mechanics during inflammation. To distinguish between effects of recirculation versus proliferation, we treated wild-type and OT-II mice with either CD62L antibody or PBS, immunized them with KLH/CFA and measured tissue properties at day 4 after immunization. Mice carrying the OT-II TCR transgene suppress more than 90% of their natural TCRs^30^, thereby eliminating a large fraction of KLH responses compared to wild-type mice. Blocked homing reduced the effective resistance in both wild-type and OT-II mice, while impaired proliferation in OT-II compared to wild-type mice only showed modest effects (Fig. 1i,j), similar with the findings under homeostasis. While viscosity was not affected, the Young’s modulus was reduced when homing was blocked in inflammation (Extended Data Fig. 1i). Thus, lymphocyte trapping is not a consequence but a cause of LN swelling. It generates an outward pressure force which is countered by the organ’s effective resistance.

### The stromal network stretches upon lymph node swelling

**Fig. 2:**
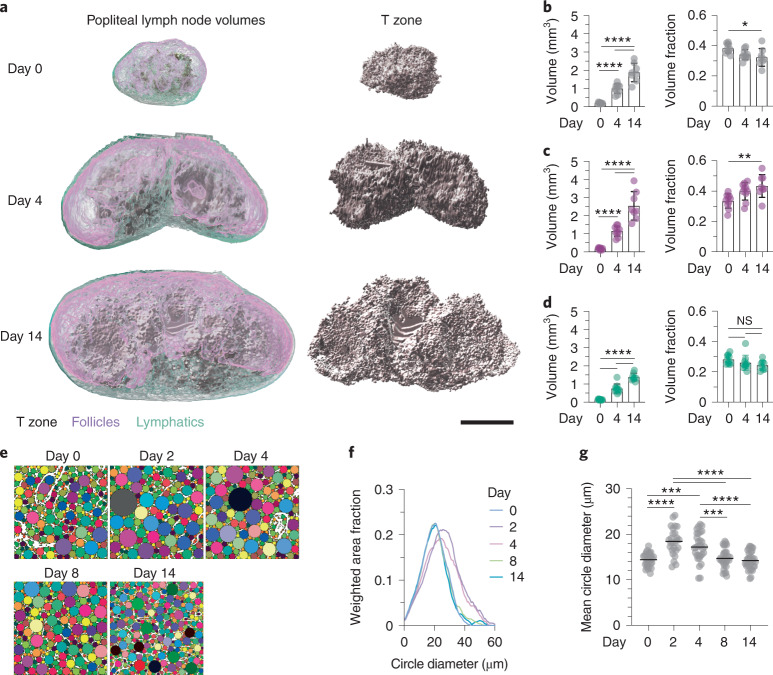
The stromal network stretches upon lymph node swelling. **a**, Representative T-zone, follicles and lymphatic compartment volumes as identified by CD3ε, B220 and LYVE-1 staining, respectively, from cleared and 3D LSFM-imaged entire popliteal LNs at homeostasis (day 0) and inflammation (days 4 and 14), visualized together and separately for the T-zone. Indentations of follicles into the underlying T-zone can be observed at all time points. Scale bar, 500 µm. **b**–**d**, Quantification of absolute and fractional volumes of T-zone (**b**; *n* = 10, 10 and 8), follicles (**c**; *n* = 10, 10 and 8) and lymphatics (**d**; *n* = 10, 10 and 8). **e**, Representative images of TRC networks gap analysis in homeostasis (day 0) and inflammation (days 2, 4, 8 and 14). **f**, Averaged and smoothed distribution of the TRC network fitted circle distributions plotted as the weighted area fraction as a function of the fitted circle diameter as measured in **e** (*n* = 28, 26, 31, 31 and 32). **g**, Quantification of the mean fitted circle diameter as in **f** (*n* = 28, 26, 31, 31 and 32). Data from **b**–**d** are shown as the mean ± s.e.m. and **g** as the mean only. Datapoints in **b**–**d** represent independent measurements of single popliteal LNs and in **g** represent the average of 10–30 analyzed consecutive optical sections of an acquired deep T-zone volume. All statistical analysis was performed using one-way ANOVA. All experiments were repeated independently (≥5 lymph nodes from ≥3 mice and ≥2 experiments) and data from **f** and **g** were pooled for each time point. For statistical details, see Supplementary Table 1. NS, not significant. *P < 0.05, **P < 0.01, ***P < 0.001, ****P < 0.0001. Source data

**Extended Data Fig. 2:**
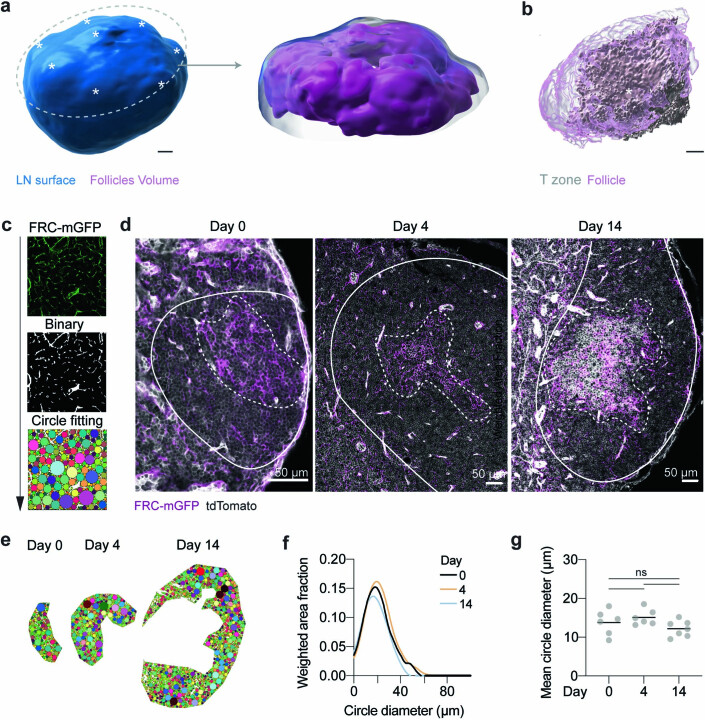
The stromal network stretches upon lymph node swelling. (**a**) 3D reconstruction of an entire homeostatic (day 0) popliteal lymph node (LN) by LSFM following clearing and staining of T-zone, follicles and lymphatic compartment volumes by CD3ε, B220 and LYVE-1 staining, respectively. LN surface and follicles are shown. Asterisks (*) indicate sites where follicles locally deform the LN’s capsule (left). Scale bar = 200 µm. 3D clipping of the dashed line encircled region (right). Follicle location with respect to capsule deformations are shown. (**b**) Example of T-zone deformation by B cell follicle (3D cropped). Asterisk (*) depicts the site at which underlying T-zone is indented by and curves around a follicle. Scale bar = 100 µm. (**c**) LN stromal network gap analysis. FRC-mGFP (top), segmented FRC network (middle), fitted circles in gaps (bottom), randomly colored, T-zone reticular cell (TRC) network in white. (**d**) Representative examples of the B cell follicle light zone (and for inflammation conditions also GC) FDC stromal network (within dashed line), and CXCL12^+^ reticular cell (CRC) stromal network (area between the dashed and solid lines) of LNs in homeostasis (day 0) and inflammation (day 4 and 14) from wild-type (WT) FRC-mGFP mice used for gap analysis as in **c**. Scale bars = 50 µm. (**e**) Representative examples of the CRC stromal network gap analysis as in **d**. (**f**) Averaged and smoothed distribution of the CRC stromal network gaps, plotted as the weighted area fraction as a function of the fitted circle diameter as in **e** (n = 5, 5, 5). (**g**) Quantification of the mean fitted circle diameter as in **e** (n = 5, 5, 5). Data from **f**, **g**, shown as mean. Datapoints in **g** represent independently measured follicles. Statistical analysis was performed using one-way ANOVA (**g**). All experiments were repeated independently (3 lymph nodes from ≥3 mice and ≥2 experiments). For statistical details, see Supplementary Information, table 1. ns, not significant. Source data

Next, we investigated which mechanical features of the LN resisted expansion. The candidate structures mediating effective resistance to swelling are the organ capsule, the FRC network and its associated ECM. We first measured how the FRC compartment adapted. Using 3D light-sheet fluorescence microscopy (LSFM), we measured the growth of T-zone, B cell follicles and lymphatics upon inflammation (Fig. 2a–d). In homeostasis, T-zone, follicles and lymphatics had relative volume fractions of 0.38, 0.34 and 0.28, respectively. These fractions remained fairly stable at day 4 of inflammation (Fig. 2b–d). At day 14, the relative volume fraction of follicles grew an additional 10%, mainly at the dispense of the T-zone (Fig. 2b–d). At the sinus interface, B cell follicles bulged into the capsule and also impressed the surrounding T-zone, suggesting that these largely ECM-devoid structures are stiffer and mechanically discontinuous from the neighboring stromal compartments (Fig. 2a and Extended Data Fig. 2a,b).

We next investigated how the stromal networks adapt to volumetric changes. To this end, we quantified gaps within the stromal networks in Ccl19-*Cre* mTmG mice, which express membrane GFP (mGFP) in FRCs (including TRCs and CXCL12^+^ reticular cells (CRCs), hereafter FRC-mGFP mice)^31,32^. A circle fitting algorithm was used to quantify the distribution of gaps from histological sections (Extended Data Fig. 2c). While no obvious disruptions of network integrity were observed, we found that TRC and CRC networks dynamically adapted over time (Fig. 2e–g and Extended Data Fig. 2d–g). At day 4 of inflammation, the TRC, but not the CRC network, widened and returned to homeostatic levels by day 14 (Fig. 2f,g and Extended Data Fig. 2f,g). These data suggest that the intact TRC network initially stretched upon swelling and subsequently remodeled to accommodate the increased numbers of immigrating and proliferating lymphocytes.

### Conduits are stretched in the swelling lymph node

**Fig. 3:**
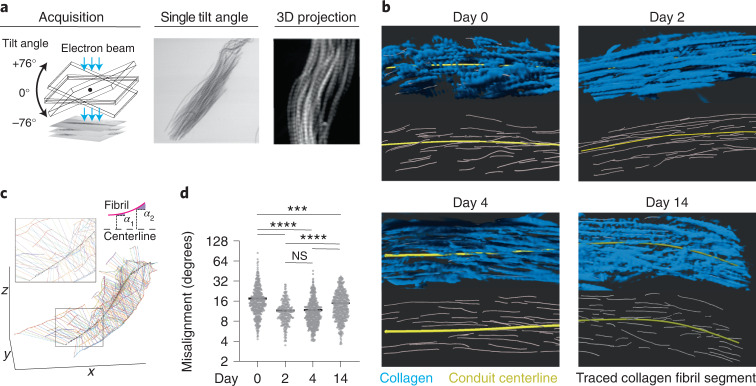
Conduits are stretched in the swelling lymph node. **a**, Schematic of STEM tomography acquisition of macerated popliteal lymph node samples (left) and images of the fibrillar collagen of T-zone conduits at a single tilt angle (middle) and a maximum intensity projection crop of a 3D conduit reconstructed from multiple tilting angles (right). **b**, Representative cropped 3D reconstructions of fibrillar collagen (blue) from macerated conduits at homeostasis (day 0) and inflammation (days 2, 4 and 14) in which the conduit centerline (yellow) and traced fibril segments (gray) are depicted. **c**, Visual representation of the conduit fibril alignment analysis of an imaged 3D conduit volume. Angles of individual fibril segments (thick colored lines) with the centerline of the conduit (dashed black line) are measured at multiple points along the fibril segment (thin colored lines) and averaged per fibril segment. *ɑ*_1_ and *ɑ*_2_ indicate measured angles. **d**, Quantification of conduit fibril alignment with centerline (*n* = 437, 244, 502 and 478). Data are shown as the mean. Datapoints represent an individual fibril segment. Statistical analysis was performed using the Kruskal-Wallis test. All experiments were repeated independently (three lymph nodes from two mice and two experiments) and data were pooled for each time point. For statistical details, see Supplementary Table 1. NS, not significant. ***P < 0.001, ****P < 0.0001. Source data

**Extended Data Fig. 3:**
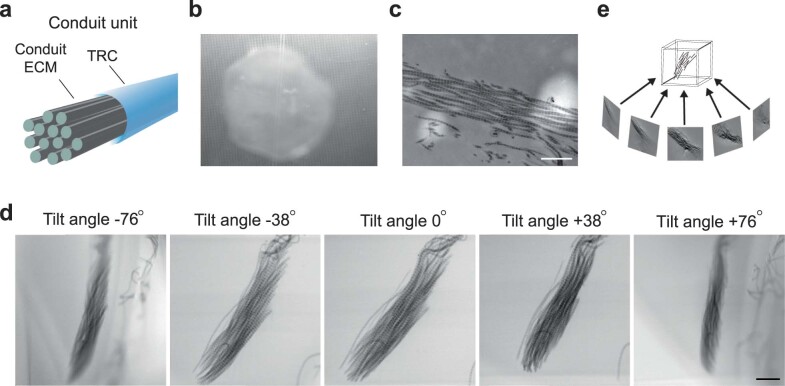
Conduits are stretched in the swelling lymph node. (**a**) Schematic illustration of the conduit unit from the T-zone. (**b**) Example of a decellularized homeostatic (day 0) popliteal lymph node (LN). (**c**) Conduit imaged by scanning transmissive electron microscopy (STEM) in which the characteristic D-period of collagen fibrils can be observed. Scale bar = 500 nm. (**d**) Examples of a T-zone conduit at different tilting angles acquired by STEM tomography. Scale bar = 500 nm. (**e**) Schematic of computed weight back projection to reconstruct a 3D volume of a conduit from differential tilting angles.

The TRC network has two principal structural components: the TRCs and the ECM conduits (Extended Data Fig. 3a). Both components have the potential to bear load and confer mechanical resistance to swelling. We quantitatively measured if and to what extent the two structures experienced mechanical forces. We started out with the ECM component and analyzed the structural organization of the conduit’s fibrillar collagen as a proxy for mechanical strain. Like in tendons and other elastic ECM structures, fibrillar alignment should increase with strain. We fixed homeostatic and reactive LNs and removed all cellular components by alkali maceration (Extended Data Fig. 3b,c). To resolve the 3D organization of individual collagen fibrils, scanning transmission electron microscopy (STEM) tomograms of T-zone conduits were acquired (Fig. 3a and Extended Data Fig. 3d,e). Measuring the misalignment of individual collagen fibrils relative to the conduit centerline reflects the extent of conduit stretching (Fig. 3b,c). We found that, compared to homeostasis (day 0), early in inflammation (day 2 to day 4) conduit collagen fibrils become progressively aligned, whereas later in inflammation (day 14) they again adopted a misaligned configuration (Fig. 3d). These results suggested that conduits stretched and bore an increased mechanical load early upon LN swelling, while at later time points, they reverted to the homeostatic state.

### T-zone reticular cell network tension increases upon lymph node swelling

**Fig. 4:**
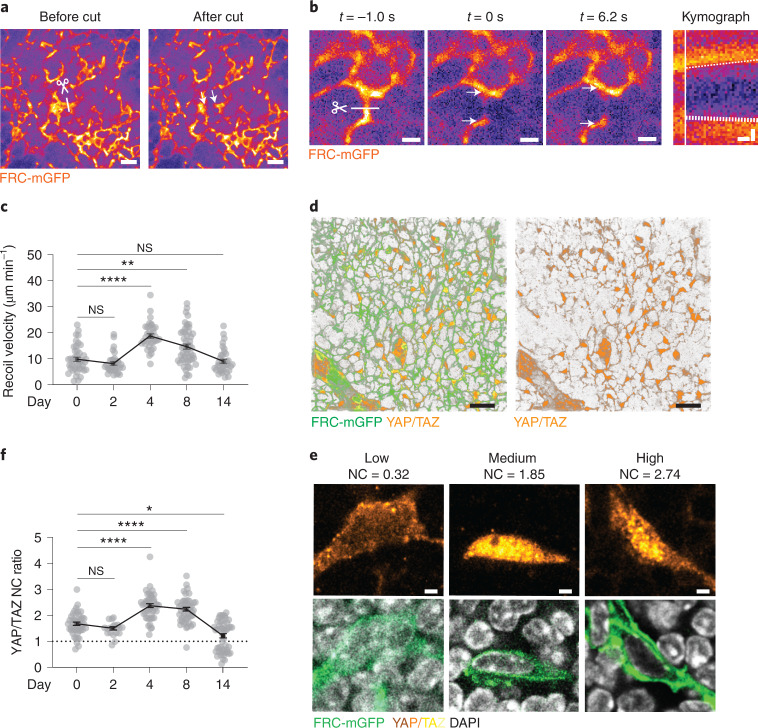
TRC network tension increases upon lymph node swelling. **a**, In vivo ultraviolet (UV) laser cut measurement of the TRC network at subcapsular IF regions where a high UV laser cuts the TRC network along 10 µm at three *z* planes after which the local recoil of the TRC network is imaged. The scissor and line indicate the cutting location and arrows indicate the recoiling FRC network. Scale bars, 20 µm. **b**, Representative example of TRC network recoil. Images depict stills from before (*t* = −1s), directly after (*t* = 0 s) and late after (*t* = 6.2 s) cutting (scale bars, 5 µm), with corresponding kymograph along the recoil axis (scale bar, *x* axis = 1 s and *y* axis = 2 µm). Scissor and line indicate cutting location and arrows show the recoiling TRC network. Dashed lines in the kymograph indicate slopes used to calculate the recoil velocity, and the vertical white line indicates the cut. **c**, Quantification of recoil velocity from kymographs as in **b** in homeostasis (day 0) and inflammation (days 2, 4, 8 and 14; *n* = 43, 33, 35, 51 and 36). **d**, 3D view of the TRC network stained for YAP/TAZ. Stack size, 20 µm. Scale bar, 20 µm. **e**, Representative examples of YAP/TAZ nuclear and cytoplasmic localization from TRCs of the deep T-zone. NC, nuclear to cytoplasmic. Scale bars, 2 µm. **f**, Quantification of YAP/TAZ NC fluorescence intensity ratio (*n* = 46, 19, 46, 48 and 50). Dashed line indicates an equal ratio. Data from **c** and **f** are shown as the mean ± s.e.m. where means are connected by a line. Datapoints in **c** represent single TRC network cuts and in **f** represent single measured TRCs. Statistical analysis was performed using the Kruskal-Wallis test. All experiments were repeated independently (≥5 lymph nodes from ≥3 mice and ≥2 experiments) and data were pooled for each time point. For statistical details, see Supplementary Table 1. NS, not significant. *P < 0.05, **P < 0.01, ****P < 0.0001. Source data

**Extended Data Fig. 4:**
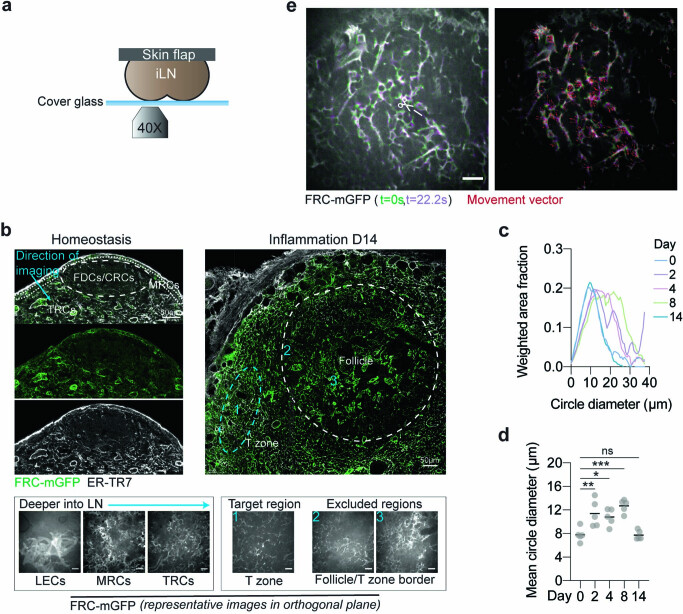
TRC network tension increases upon lymph node swelling. (**a**) Schematic of *in vivo* laser cutting in inguinal lymph nodes (iLNs) of FRC-mGFP mice. (**b**) Subcapsular T-zone reticular cell (TRC) network is imaged at interfollicular (IF) regions (blue arrow). Homeostatic (day 0) and inflammation (day 14) are shown. mGFP positive lymphatic endothelial cells (LECs), marginal reticular cells (MRCs) and TRCs are encountered while gradually focusing into the LN, while follicular dendritic cell (FDC) and CXCL12^+^ reticular cells (CRCs) regions are avoided. The T-zone at IF sites is identified by regular network morphology and bright fluorescent reporter intensity (1), compared to the more irregular and dimmer CRC (2) and dense and bright FDC (3) stromal networks found in the largely ECM-free (ER-TR7 negative) follicles. White dashed lines indicate the outline of follicles, and white dense spaced lines indicate the subcapsular region where MRCs are found (annotated in homeostasis). The area enclosed by the blue dashed line shows a typical area targeted for TRC laser cutting in the orthogonal plane. Scale bars: top = 50 µm, bottom = 20 µm. (**c**) Averaged and smoothed distribution of the subcapsular TRC network as analyzed by circle fitting gap analysis of homeostatic (day 0) and inflamed (day 2, 4, 8, 14) LNs from FRC-mGFP mice, plotted as the weighted area fraction as a function of the fitted circle diameter (n = 5, 5, 5, 5, 5). (**d**) Quantification of the mean fitted circle diameter as in **c** (n = 5, 5, 5, 5, 5). (**e**) Example of force propagation in the TRC network following a UV laser cut. Time point directly after cutting (t = 0 s) and late after cutting (t = 22.2 s) overlapped (left). Scissor and white line indicate the laser cut location. Scale bar = 20 µm. Overlay of movement vectors (red arrows) derived from particle imaging velocimetry analysis (right). Data from **c** shown as mean and **d** shown as mean ± s.e.m. Datapoints in **d** represent independently analyzed TRC network images from LNs used in laser cutting experiments. Statistical analysis was performed using one-way ANOVA (**d**). All experiments were repeated independently (3 mice and ≥2 experiments). For statistical details see Supplementary Information, table 1. NS, not significant. *P < 0.05, **P < 0.01, ***P < 0.001. Source data

We next investigated the role of TRCs in the stretching response. To study if the change in conduit conformation was mirrored by the tension state of the TRC network, we directly measured TRC network tension by in situ laser ablation and recoil analysis^33,34^. To this end, the TRC network in FRC-mGFP mice was imaged under the capsule at interfollicular (IF) sites where network dynamics were similar to the deep paracortex (Extended Data Fig. 4a–d, Fig. 2g and Supplementary Movie 2). Cutting individual strands of the 3D network caused immediate recoil of TRCs, followed by a local repositioning of the adjacent network (Fig. 4a,b, Extended Data Fig. 4e and Supplementary Movie 3). At days 4 and 8 of inflammation, tension within the TRC network had almost doubled compared to that at homeostasis (day 0) but was restored to homeostatic levels at day 14 (Fig. 4c and Supplementary Movie 4).

As a proxy for the cellular mechanosensing response, we next measured nuclear versus cytoplasmic localization of the transcription factors YAP and TAZ, which are well-established downstream responses of cytoskeletal tension^35^. TRCs and endothelial cells stained positive for YAP and TAZ, while leukocytes were devoid of signal (Fig. 4d,e). The nuclear/cytoplasmic ratio of YAP and TAZ (YAP/TAZ NC ratio) in TRCs remained stable from day 0 to day 2 of inflammation but increased at day 4 and day 8 (Fig. 4f), indicating that TRCs experience increased cytoskeletal tension during LN swelling. The YAP/TAZ NC ratio decreased after TRC tension peaked (Fig. 4c,f), thereby faithfully recapitulating the tension as measured by laser cutting. At week 2 after immunization, we observed a large population of TRCs that had a negative YAP/TAZ NC ratio (Fig. 4f), suggesting that those cells were completely shielded from active tension. These data suggested that TRC tension increased upon LN swelling and restored to homeostatic conditions 2 weeks after immunization.^24,25^

### T-zone reticular cells undergo distributed clonal expansion

**Fig. 5:**
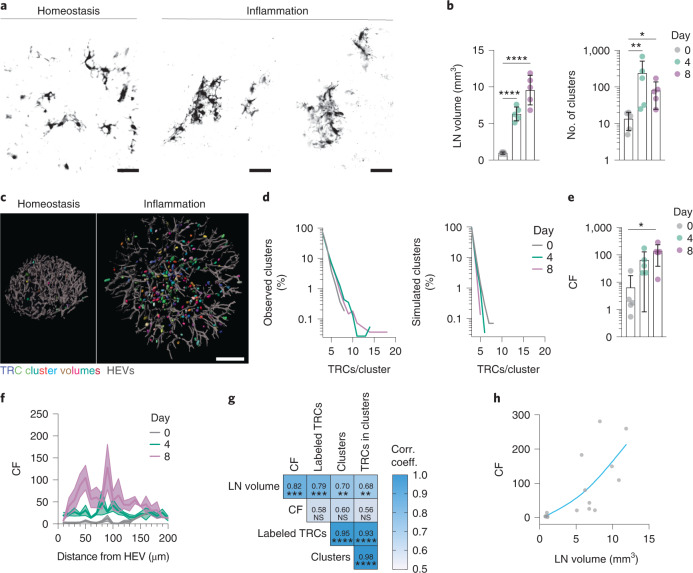
T-zone reticular cells undergo distributed clonal expansion. **a**, High-resolution confocal volumes of MADM sparse labeled TRCs in homeostasis (day 0) and TRC clusters in inflammation (day 8). Scale bars, 40 µm. **b**, Quantification of LN volume (left; *n* = 5, 5 and 5) and number of clusters (right; *n* = 5, 5 and 5) of light-sheet images from cleared popliteal lymph nodes of Ccl19-*Cre* MADM-7^GT/TG^ mice in homeostasis (day 0) and inflammation (days 4 and 8). **c**, Representative images of TRC cluster volumes (randomly colored) and HEVs of an entire LN in homeostasis (day 0) and inflammation (day 4) from experiments as in **b**. Scale bar, 200 µm. **d**, Frequency distribution in percentages of TRC per cluster found in observed and simulated data from experiments as in **b**. Data are depicted as the mean (*n* = 5, 5 and 5). **e**, Quantification of the CF per LN from experiments as in **b** (*n* = 5, 5 and 5). **f**, CF plotted as a function of the distance from the nearest HEV from experiments as in **b**. Data are depicted as the mean ± s.e.m. (*n* = 5, 5 and 5). **g**, Correlation matrix of paired variables assessed in the cluster analysis from experiments as in **b** (*n* = 15). *P* values are given and the correlation coefficients are color coded. **h**, CF plotted as a function of the LN volume from experiments as in **b** (*n* = 15). A spline fit was plotted through the datapoints. Data from **b** and **e** are depicted as the mean ± s.d. Datapoints from **b** and **e** represent a single analyzed LN. Statistical analysis was performed using one-way ANOVA (**b**; left), Kruskal-Wallis test (**b**; right, **e**) and two-tailed Spearman correlation (**g**). All experiments were repeated independently (≥3 mice and ≥2 experiments). For statistical details, see Supplementary Table 1. **P < 0.01, ***P < 0.001, ****P < 0.0001. Source data

**Extended Data Fig. 5:**
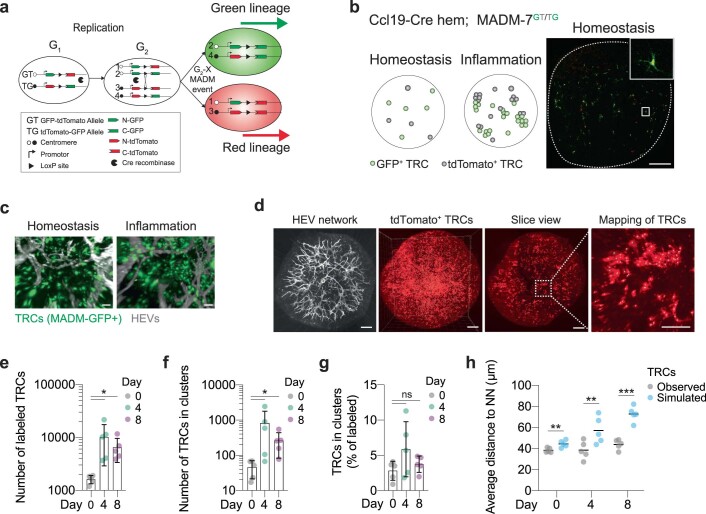
TRCs undergo distributed clonal expansion. (**a**) Schematic of the mosaic analysis with double markers (MADM) labeling principle. Rare interchromosomal recombination in the G2 cell cycle phase following x-segregation of chromosomes labels T-zone reticular cells (TRCs) with either a cytoplasmic tdTomato or GFP. (**b**) Schematic of the sparse mosaic analysis with double markers (MADM) labeling approach for TRC cluster analysis in popliteal lymph nodes (LNs) from Ccl19-*Cre* MADM-7^GT/TG^ mice in homeostasis (day 0) and inflammation (day 4 and 8) (left). Sparse labeling of TRCs in a histological section of a homeostatic LN (right). Scale bar = 200 µm. (**c**) 3D fluorescent intensity cropped images from light-sheet fluorescent microscopy of entire lymph nodes for which MADM-GFP labeled TRCs and *in situ* labeled high endothelial venules (HEVs) by PNAd-ATTO647n Ab are shown, as in **b**. Scale bars = 20 µm. (**d**) Example of the labeling of HEVs and mapping of MADM-labeled TRCs (only tdTomato^+^ TRCs are shown) at inflammation (day 4) as in **c**. The enlarged image depicts the mapping of individual TRCs by a grey sphere at the center of each cell. Scale bars = 200 µm. (**e**) Quantification of number of MADM-labeled TRCs as in **c** (n = 5, 5, 5). (**f**) Quantification of number of MADM-labeled TRCs found in clusters as in **c** (n = 5, 5, 5). (**g**) Quantification of numbers of MADM-labeled TRCs found in cluster as percentage of total MADM-labeled TRCs as in **c** (n = 5, 5, 5). (**h**) Quantification of the average distance to the nearest neighbor (NN) of both observed and simulated TRCs as in **c** (n = 5, 5, 5, 5, 5, 5). Data from **d**, **f**, **g** shown as mean ± s.d. and **h** shown as mean. Datapoints in **e**, **f**, **g**, **h** represent independently analyzed LNs. Statistical analysis was performed using Kruskal-Wallis test (**e**, **f**, **g**) and paired two-tailed t test. All experiments were repeated independently (≥3 mice and ≥2 experiments). For statistical details see Supplementary Information, table 1. NS, not significant. *P < 0.05, **P < 0.01, ***P < 0.001. Source data

To test how the TRC network expanded, remodeled and reestablished its typical configuration, we devised an approach to map the spatiotemporal expansion of the TRC network in situ. We used a sparse clonal labeling approach named mosaic analysis with double markers (MADM)^36–38^. MADM labeling results from rare interchromosomal mitotic recombination driven by Cre-loxP sites (Extended Data Fig. 5a). Two reciprocally split GFP and tdTomato genes (GT and TG) on identical loci of homologous chromosomes are used to create *trans*-heterozygous offspring (GT/TG). Interchromosomal recombination can take place in the G_2_ phase, restores functional GFP and/or tdTomato expression and thereby irreversibly labels the lineage. To trigger recombination specifically in TRCs, we used the Ccl19*-Cre* transgene^31^ and generated Ccl19-*Cre* MADM-7^GT/TG^ mice, which were immunized by KLH/CFA footpad injection (Extended Data Fig. 5b). Homeostatic (day 0) and reactive LNs (day 4 and 8) were cleared and imaged by 3D LSFM. Prominent clusters of TRCs emerged in reactive LNs, while such clusters were rarely observed at day 0 (Fig. 5a, Extended Data Fig. 5c and Supplementary Movie 5), suggesting that individual TRC clones expanded following immunization and that daughter TRCs stay close to their precursor. Quantitative analysis of TRCs using a density-based spatial clustering of applications with noise^39^ (Extended Data Fig. 5d) indicated the number and size of TRC clusters were significantly increased in reactive LNs (day 4 and day 8) compared to homeostasis (day 0; Fig. 5b,c and Extended Data Fig. 5e–g). We defined the cluster factor (CF) as the number of TRCs in observed clusters versus the in silico generated random distributions (for example, a CF of 100 indicates that 100 times more cells are found in clusters than by chance alone). TRCs in reactive LNs (days 4 and 8) formed more and larger clusters compared to homeostasis (day 0; Fig. 5d,e and Extended Data Fig. 5h) and cluster size distribution was exponential (Fig. 5d), as is expected from a stochastically dividing precursor population^40^. We found an average CF of 6 in homeostatic conditions (day 0) and 65 and 137 for day 4 and day 8 after immunization, respectively (Fig. 5e), indicating that proliferating TRCs formed clusters in the swelling LN. Because de novo TRCs can derive from perivascular fibroblasts in the developing spleen^41,42^, we plotted the CF as a function of the distance from HEVs, but found no enrichment at these sites (Fig. 5f). To better understand the relationships between the measured parameters, we created a correlation matrix (Fig. 5g), which indicated that TRCs expanded in randomly distributed clusters and that TRC growth correlated with LN volume (Fig. 5h). Together, these data suggest that TRCs could expand in response to local signals independent of their localization.

### Talin1 is required for T-zone reticular cell mechanosensing

**Fig. 6:**
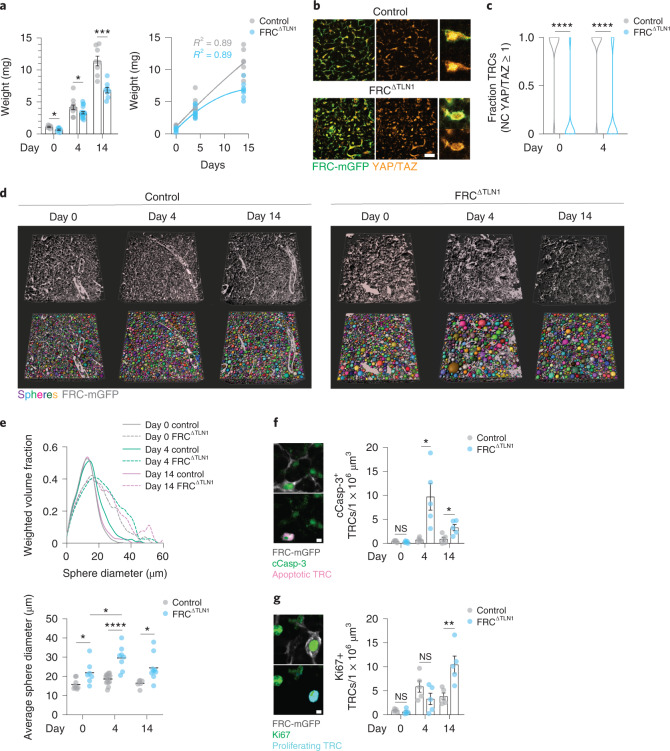
Talin1 is required for T-zone reticular cell mechanosensing. **a**, Quantification of LN weights in homeostasis (day 0) and inflammation (days 4 and 14) in littermate control and FRC^ΔTLN1^ mice (left, *n* = 6, 7, 12, 19, 8 and 8) and same data fitted by nonlinear regression (right). **b**, Representative TRC networks from littermate control and FRC^ΔTLN1^ mice in homeostasis (day 0) and at inflammation (day 4) stained for YAP/TAZ. Scale bars, 10 µm. **c**, Violin plots showing quantification of YAP/TAZ localization as in **b** (*n* = 35, 84, 152 and 134). **d**, Representative images of 3D TRC network analysis of LNs from FRC-mGFP control and FRC^ΔTLN1^-mGFP mice in homeostasis (day 0) and inflammation (days 4 and 14). Fitted spheres are randomly colored. Imaged stack size, 100–300 µm. Scale bars, 50 µm. **e**, Quantification of TRC network analysis as in **d**. Average weighted volume fraction plotted as function of the sphere diameter (top; *n* = 9, 7, 15, 8, 6 and 9). Average sphere diameter (bottom). **f**, Quantification of cCasp-3^+^ TRCs in LNs from FRC-mGFP control and FRC^ΔTLN1^-mGFP mice in homeostasis (day 0) and inflammation (days 4 and 8; *n* = 5, 5, 5, 5, 5 and 5). Images show the identification of an apoptotic TRC. Scale bar, 3 µm. **g**, Quantification of Ki67^+^ TRCs as in **f** (*n* = 5, 5, 5, 5, 5 and 5). Images show the identification of a proliferating TRC. Scale bar, 3 µm. Data from **a**, **c**, **e** (bottom), **f** and **g** are shown as the mean ± s.e.m. and **e** (top) as the mean. Datapoints in **a** represent independently measured LNs, and those in **e**–**g** represent independently measured TRC network volumes. Statistical analysis was performed using unpaired two-tailed *t*-test (**a**; left, **f** and **g**), two-tailed Fisher’s exact test (**c**) and one-way ANOVA (**e**; ln(*y*) transformed). All experiments were repeated independently (≥3 lymph nodes from ≥2 mice and ≥2 experiments) and data from **c** were pooled for each condition. For statistical details, see Supplementary Table 1. NS, not significant. *P < 0.05, **P < 0.01, ***P < 0.001, ****P < 0.0001. Source data

**Extended Data Fig. 6:**
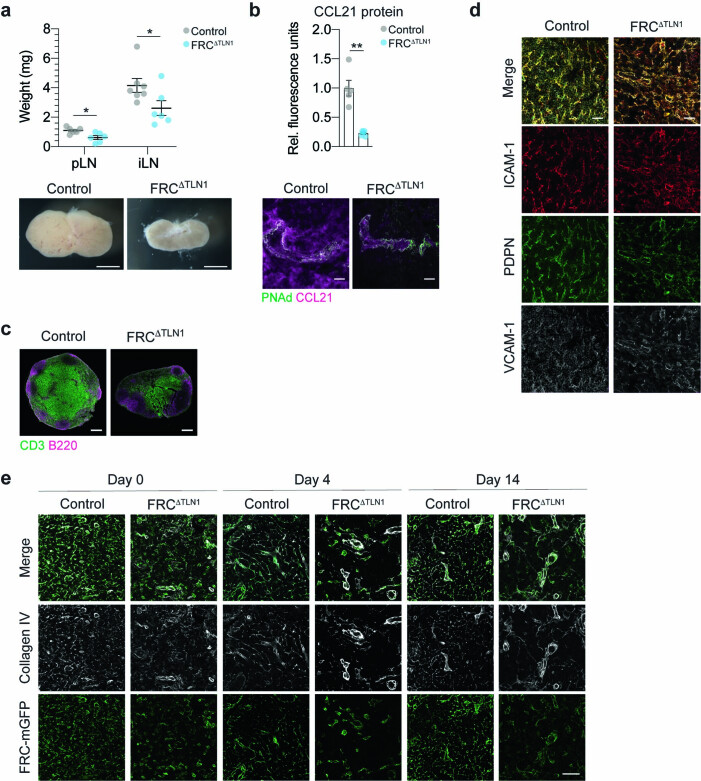
Talin1 is required for TRC mechanosensing. (**a**) Quantification of homeostatic (day 0) popliteal and inguinal lymph node (LN) weights from littermate control and FRC^ΔTLN1^ mice. Images show representative inguinal LNs from littermate control and FRC^ΔTLN1^ mice. Scale bars = 1 mm. (**b**) Quantification of homeostatic (day 0) T-zone CCL21 protein as measured *in situ* by fluorescent intensity following staining for CCL21 in littermate control and FRC^ΔTLN1^ mice. Representative images for littermate control and FRC^ΔTLN1^ mice in which CCL21 chemokine and high endothelial venule (HEVs) stained by PNAd Ab are shown. Scale bars = 20 µm. (**c**) T-zone and B cell follicles of homeostatic (day 0) popliteal LNs from littermate control and FRC^ΔTLN1^ mice stained for CD3ε (T cells) and B220 (B cells). Scale bars = 200 µm. (**d**) ICAM-1, PDPN, VCAM-1 and merged staining on histological sections of homeostatic (day 0) popliteal LN T-zones from control and FRC^ΔTLN1^ mice. Scale bars = 20 µm. (**e**) Representative histological images of T-zone reticular cell (TRC) networks from homeostatic (day 0) and inflamed (day 4 and 14) LNs from FRC-mGFP and FRC^ΔTLN1^-mGFP mice stained for collagen IV. Scale bar = 50 µm. Data from **a** shown as mean and **b** as mean ± s.e.m. Datapoints in **a**, **b** represent independently analyzed LNs. Statistical analysis was performed using unpaired two-tailed t test (**a**) and two-tailed Mann-Whitney test (**b**). All experiments were repeated independently (≥3 mice and ≥2 experiments). For statistical details see Supplementary Information, table 1. *P < 0.05, **P < 0.01. Source data

Mechanical forces could be a feedback parameter regulating the TRC network growth. Mechano-coupling of fibroblasts to their underlying matrix is mediated by integrins and their associated intracellular force-sensitive adaptor Talin^43^. FRCs express both TLN1 (Talin1) and TLN2 (Talin2) isoforms, which play nonredundant roles in integrin activation and force transduction^44^. We generated Ccl19*-Cre* *Talin1*^fl/fl^ (FRC^ΔTLN1^) mice, in which Talin1 was selectively deleted in FRCs, and also crossed them with mTmG mice, to obtain FRC^ΔTLN1^-mGFP mice, which expressed mGFP specifically in FRCs. Peripheral LNs of nonimmunized FRC^ΔTLN1^ mice were smaller compared to Ccl19*-Cre* *Talin1*^fl/+^ littermate controls (Extended Data Fig. 6a) and the expression and secretion of the chemokine CCL21 by TRCs, but not HEVs, were decreased compared to littermate controls (Extended Data Fig. 6b). While the T cell zone of the FRC^ΔTLN1^ LNs appeared smaller compared to littermate controls (Extended Data Fig. 6c), the podoplanin^+^ TRCs of FRC^ΔTLN1^ mice formed a regularly interconnected network and expressed the adhesion molecules ICAM-1 and VCAM-1 (Extended Data Fig. 6d), suggesting they differentiated normally and that the basic organization and differentiation of the Talin1-deficient TRC network in the FRC^ΔTLN1^ mice was maintained. The swelling of LNs in immunized FRC^ΔTLN1^ mice at days 1–4 after immunization was comparable to that seen in littermate controls (Fig. 6a), with a ~tenfold increase in LN weight at day 14 after immunization compared to day 0 (Fig. 6a), suggesting that lymphocyte influx and proliferation still occurred in the Talin1-deficient TRC network and that the FRC^ΔTLN1^ mice were suitable to test whether mechanosensing in TRCs was required for network adaptation. In the FRC^ΔTLN1^-mGFP mice, based on the YAP/TAZ NC ratio, almost no TRCs showed nuclear localization of YAP/TAZ under homeostatic (day 0) or reactive conditions (day 4; Fig. 6b,c), indicating that Talin1-deficient TRCs lost their mechanosensitivity.

To assess the functional consequences of loss of TRC mechanosensing for the TRC network integrity during LN swelling, we performed in situ network analysis. Three-dimensional volumes of TRC networks were acquired from cleared thick vibratome LN slices and network spacing was quantified using a 3D sphere filling algorithm (Fig. 6d,e). Under homeostatic conditions (day 0), the TRC network in the FRC^ΔTLN1^-mGFP mice was widened compared to that seen in FRC-mGFP controls but structurally intact (Fig. 6d). Upon immunization, the Talin1-deficient TRC network integrity was severely compromised, showing large TRC-free gaps that were most apparent at day 4 and partly recovered by day 14 after immunization (Fig. 6d,e and Extended Data Fig. 6e), suggesting that the TRC network in FRC^ΔTLN1^-mGFP mice failed to adapt to organ swelling and partly disintegrated or ruptured. Staining for cleaved caspase 3 (cCasp-3) and Ki67 to identify apoptotic and proliferating TRCs, respectively, showed little apoptotic or proliferating TRCs in FRC^ΔTLN1^-mGFP and FRC-mGFP control mice under homeostatic conditions (Fig. 6f,g). At day 4 after immunization, the number of apoptotic cCasp-3^+^ TRCs per volume increased significantly in FRC^ΔTLN1^-mGFP mice compared to FRC-mGFP controls, while the number of proliferating Ki67^+^ TRCs was similar (Fig. 6f,g). At day 8, apoptotic cCasp-3^+^ TRCs per unit volume were still larger in FRC^ΔTLN1^-mGFP compared to FRC-mGFP controls, while proliferating Ki67^+^ TRCs were increased (Fig. 6f,g). These data indicate that compromised mechanosensing caused a severe dysregulation in survival and proliferation of the TRC compartment, leading to a loss of network integrity.

### Capsule fibrosis constrains late lymph node expansion

**Fig. 7:**
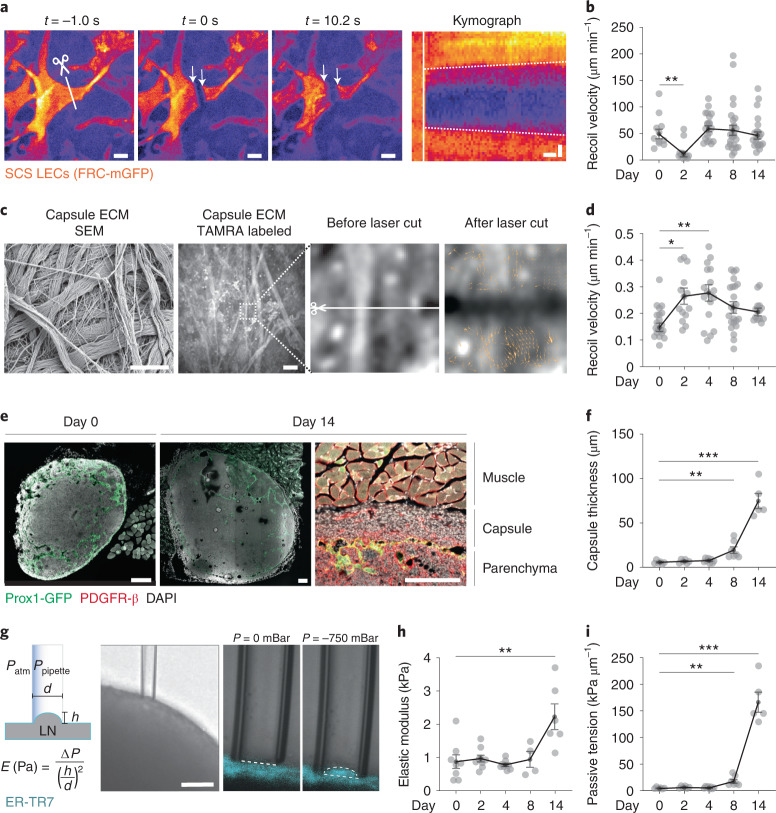
Capsule fibrosis constrains late lymph node expansion. **a**, Representative images from in vivo laser cut experiments of subcapsular sinus LECs before (*t* = −1s), directly after (*t* = 0 s) and late after (*t* = 10.2 s) cutting (scale bars, 5 µm), with corresponding kymograph along the recoil axis (scale bar, *x* = 1 s and *y* = 2 µm). The scissor and line indicate the location of the cut and arrows indicate the recoiling cell. Dashed lines in the kymograph indicate slopes and the vertical white line shows the cut. **b**, Quantification of experiments as in **a** during homeostasis (day 0) and inflammation (days 2, 4, 8 and 14; *n* = 11, 12, 20, 25 and 22). **c**, UV laser cut experiment on TAMRA-labeled capsule ECM of explanted LNs in homeostasis (day 0) and inflammation (day 2, 4, 8 and 14), which recapitulate ECM from scanning electron microscopy (SEM) imaging. Recoil displacement is depicted by orange vectors. Scale bars, SEM image = 1 µm and fluorescence image = 20 µm. **d**, Quantification of recoil velocities in **c** (*n* = 20, 14, 18, 24 and 14). **e**, Representative examples of LN capsules from Prox1-GFP mice in homeostasis (day 0) and inflammation (day 14), stained for PDGFR-β (only shown on right) and counterstained with DAPI. Scale bars, 200 µm. **f**, Quantification of capsule thickness as in **e** (*n* = 6, 6, 8, 7 and 5). **g**, Capsule stiffness measurements of homeostatic (day 0) and inflamed (days 2, 4, 8 and 14) explanted popliteal LNs labeled with ERT-R7 antibody. Scale bar, 50 µm. **h**, Quantification of capsule stiffness as in **g** (*n* = 8, 9, 9, 4 and 6). **i**, Quantification of passive tension (*n* = 6, 6, 8, 7 and 5). Data from **b**, **d**, **f**, **h** and **i** are shown as the mean ± s.e.m. Datapoints in **b** and **d** represent independent cuts, and data in **f**, **h** and **i** show independently measured LNs. Statistical analysis was performed using the Kruskal-Wallis test (**b**, **f**, **h** and **i**) and one-way ANOVA (**d**). All experiments were repeated independently (≥4 lymph nodes from ≥4 mice and ≥2 experiments) and data from **b** and **d** was pooled for each condition. For statistical details, see Supplementary Table 1. *P < 0.05, **P < 0.01, ***P < 0.001. Source data

**Extended Data Fig. 7:**
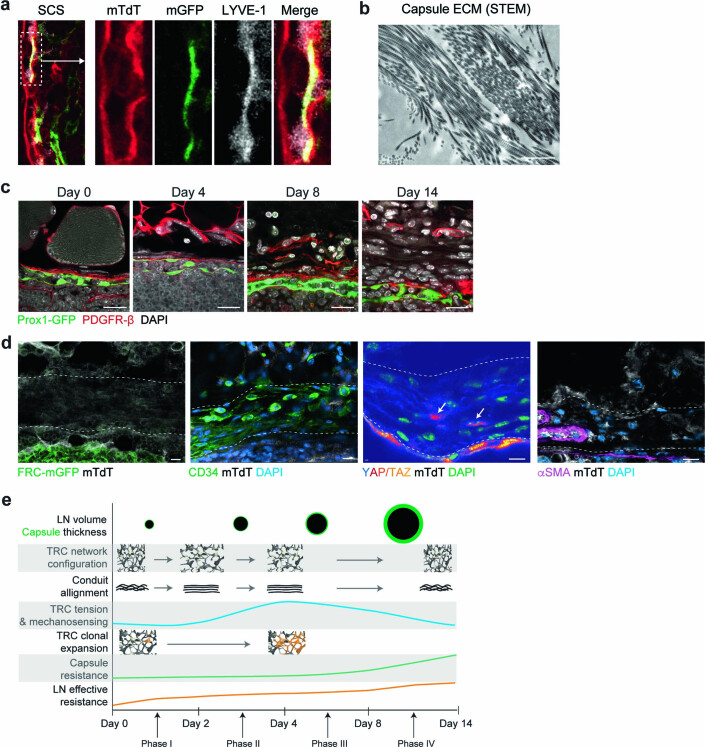
Capsule fibrosis constrains late lymph node expansion. (**a**) Crop of a representative histological image of a FRC-mGFP mouse derived homeostatic (day 0) inguinal lymph node (LN) stained for LYVE-1. mGFP is expressed specifically in cells with *Cre*-recombinase activity and membrane-bound tdTomato (mTdT) in all other cells. LYVE-1^+^ floor lymphatic endothelial cells (fLECs) are sparsely labeled by mGFP in the subcapsular sinus (SCS). (**b**) Capsule extracellular matrix (ECM) from an alkali-macerated popliteal LN from a wild-type (WT) mouse imaged by scanning transmissive electron microscopy (STEM). Scale bar = 5 µm. (**c**) Representative histological images of LN capsules from Prox1-GFP mice in homeostasis (day 0) and inflammation (day 4, 8 and 14) in which LECs are labeled by a cytoplasmic GFP. Mesenchymal cells are stained for PDGFR-β, and nuclei are counterstained with DAPI. Scale bars = 20 µm. (**d**) Characterization of capsular fibroblasts in inflamed (day 14) popliteal LNs from a FRC-mGFP (left panel; FRCs labeled with mGFP and all other cells with mTdT) or mTmG (other panels; all cells are labeled with mTdT) mouse of which the latter was stained for CD34, YAP/TAZ or αSMA and counterstained with DAPI. Areas between dashed lines indicate the capsule. Arrows indicate YAP/TAZ positive fibroblast nuclei in the capsule. Scale bars = 10 µm. (**e**) Schematic of mechanical dynamics and the multi-tier model of the swelling LN. Phase I: LN growth with T-zone reticular cell (TRC) relaxation and stretching. Phase II: LN growth with increasing TRC tension and TRC network expansion. Phase III: LN growth with decreasing TRC tension and further TRC network expansion. Phase IV: LN growth and TRC network expansion with decreasing TRC tension, capsule thickening and increasing strong resistance to growth.

Although the TRC network reached a ‘new equilibrium’ at week 2 after immunization, when it readopted its homeostatic configuration, effective resistance remained high at this late time point. To test if another structure contributed to the force balance, we used in situ laser ablation to investigate the structural and mechanical properties of the LN capsule, which can be divided into two components: a floor that includes floor lymphatic endothelial cells (fLECs), which are sparsely labeled in FRC-mGFP mice (Extended Data Fig. 7a), and a roof that consists of ECM with embedded fibroblasts (Extended Data Fig. 7b–d). In vivo laser cutting of fLECs showed high basal tension during homeostasis (Fig. 7a,b), which transiently dropped at day 2 after immunization and reverted to homeostatic levels at day 4 (Fig. 7b), while roof tension showed a single increase at day 2 after immunization compared to homeostasis (day 0; Fig. 7c,d). These observations, indicating the absence of a continuous rise in active tension on the capsule floor and roof after immunization, suggested that these components were being continuously remodeled to keep up with the ongoing volumetric increase of the swelling LN. Histology of the LN capsule in Prox1-GFP mice, in which the cytoplasm of all LECs is labeled with GFP (Fig. 7e and Extended Data Fig. 7c), indicated that the capsule thickness remained unchanged at days 1–4 after immunization, but increased ~14-fold at days 8–14, forming a dense fibrotic layer between the parenchyma and surrounding adipose and muscle tissue (Fig. 7e,f). Further histological characterization showed that the fibroblasts in the thickened capsule were not labeled in FRC-mGFP mice, were positive for CD34 (ref. ^16^), had occasional YAP/TAZ^+^ nuclei and did not express α-smooth muscle actin (αSMA; Extended Data Fig. 7d), indicating that these fibroblasts were phenotypically distinct from FRCs.

To ask if the remodeling of the capsule resulted in changes in its mechanical properties, wild-type LN capsules labeled with an antibody to the fibroblast marker ER-TR7 were aspirated with a micropipette and an effective Young’s modulus (stiffness) of the capsule was derived from the aspiration depth measurements using Laplace’s law (Fig. 7g). The Young’s modulus of the capsule remained stable over days 1–8 after immunization, but doubled at day 14 (Fig. 7h). By multiplying the capsule thickness and Young’s modulus of the capsule, we derived the passive capsule tension, which is a measure of the amount of force necessary to enlarge the whole thickness of the capsule by a certain length. The passive tension of the capsule showed a substantive increase (3.8-fold) from day 0 to 8 of inflammation and kept rising to a 35-fold increase at day 14 of inflammation (Fig. 7i). These data indicate that during a sustained immune response, tension dissipated from the remodeling TRC network at day 4 to day 14, while the capsule remodeled and became thicker, stiffer and more resistant to swelling at day 8 to day 14, establishing a new force equilibrium within the organ, which resisted further swelling (Extended Data Fig. 7e).

## Discussion

Here we established that reactive swelling of the LN was a multitier process controlled by mechanical feedback. This allowed the organ to expand in a stepwise controlled fashion, without compromising its delicate architecture.

Upon inflammation, accumulating lymphocytes inflated the node and initially stretched the TRC network. This is in line with previous findings that TRCs show an early and transient increase in cell size^21^. Stretching put tension on the ECM conduit, as revealed by a straightened configuration of its ECM fibrils. B cell stroma did not show signs of stretching, indicating that already early in inflammation the largely ECM-free architecture of the follicular network grew proportionally with the lymphocyte compartment. Our laser cutting experiments and the kinetics of nuclear shuttling of YAP/TAZ indicated that, compared to their ECM conduit, TRCs experienced cytoskeletal tension with a time delay of 2 days. This confirms findings that, early in inflammation, an interaction between C-type lectin-like receptor 2 (CLEC-2) on activated dendritic cells (DCs) and podoplanin on TRCs relaxes actomyosin contractility of TRCs and thereby allows the stromal network to stretch^24,25^. Such a transient relaxation explains why the tension increase of the ECM conduit preceded the tension increase of TRCs. It also implies that the TRC cytoskeleton only experiences significant tension once the DC-mediated relaxation signals fade after 3 to 4 days, which is the time window in which TRCs increase their expression of αSMA^21^. We found a similar drop in tension around day 2 in PDPN^+^ LECs of the subcapsular sinus floor, indicating that transmigrating DCs might also influence the mechanical state of this stromal population via the CLEC-2–PDPN axis^45,46^.

While the TRC network used its intrinsic elasticity to accommodate short-term volumetric changes, sustained strain on the TRC cytoskeleton triggered the next stage of LN swelling, which was characterized by actual growth and structural remodeling of the network. Our results in Talin1-deficient TRCs support the idea that adhesion-dependent mechanosensing was a critical feedback parameter that locally controlled growth and survival of the network, so that it reverted to its typical geometry, while increasing in size. A critical prerequisite of a model where TRC mechanosensing locally controls network remodeling is that the TRC responsiveness is not restricted to specific niches, but rather distributed throughout the organ. Our clonal analyses showed that this was indeed the case. In line with mechanics being a critical control parameter, mice with a gain-of-function mutation in the mechanosensitive YAP/TAZ pathway showed fibrotic LNs with impaired FRC differentiation^47^ and blockade of β1 integrin triggered FRC apoptosis in swelling, but not in homeostatic, LNs^48^.

Beyond 1 week of structural adaptation, the TRC network of the now massively enlarged LN seemed to reach a new ‘mechanical equilibrium’, as indicated by gap analysis, ECM alignment, tension measurements and YAP/TAZ translocation. Nevertheless, bulk mechanical properties did not return to homeostatic levels, but rather showed an elevated effective resistance, indicating that another structure countered further organ expansion. We identified the capsule as the responsible stromal element for elevated resistance to swelling from day 8 to day 14, during which its thickness and mechanical strength were massively increased. Although capsule fibrosis is a characteristic histopathological descriptor of reactive LNs^49^, its mechanistic contributions remain to be explored. While our work sheds some light on LN swelling, it is still unknown how the expansion process is reverted. Here, our observation that a large population of TRCs lost nuclear YAP/TAZ at week 2 of inflammation might mark the beginning of an involution process, where decreasing lymphocyte numbers and a concomitant drop in TRC tension initiate a reductive network remodeling.

The proposed multitier model of LN swelling implicates a succession of checkpoints and can be adapted to very different types of swelling scenarios. Transient swelling, as occurs during circadian fluctuations, might stretch the network, but is unlikely to cause structural remodeling. On the contrary, sustained immune responses with massive lymphocyte trapping and germinal center reactions might rely on a fibrotic strengthening of the capsule to limit excessive expansion of the organ. Our findings demonstrate that mechanical forces are decisive feedback parameters orchestrating LN swelling at the cellular and organ scales.

## Methods

### Mice

All animal experiments were performed in accordance with the Austrian law for animal experiments. Permission was granted by the Austrian Federal Ministry of Science, Research and Economy (identification codes: BMWFW 66.018/0010-WF/V/3b/2016 and 66.018/0027-WF/V/3b/2014). Experimental plans and treatment regimens were selected in consultation with IST Austria Ethical Committee. Mice were bred and maintained at the local animal facility or purchased from Charles River and maintained at the local animal facility in accordance with IST Austria Ethical Committee taking into account national and European guidelines. OT-II (stock no. 004194) and mTmG (stock no. 007576), were obtained from JAX. Ccl19-*Cre* mice have been described previously^31^. MADM-7 (ref. ^38^), Talin1-floxed^50^ and Prox1-GFP^51^ mice were provided by S. Hippenmeyer and D. Critchley. All mice are on a C57BL/6J background, with exception of MADM-7, which have a CD-1 background. Mice of both sexes between the ages of 6 and 20 weeks were used for experiments and were randomly assigned to treatment and control groups. For immunization, KLH protein was dissolved in PBS to 5 mg ml^−1^ and then mixed at a 1:1 ratio with CFA (both Sigma-Aldrich) upon which 40 μl of the immunization mixture was injected into the footpads and flanks of draining popliteal and inguinal LNs. LNs were collected after various time points up to 2 weeks after immunization to be used for histology or explant experiments or used for in vivo imaging experiments. For LN cellularity manipulation experiments, mice were intravenously injected with 100 μg CD62L antibody (MEL14, BioXCell) and control mice with PBS alone. For steady-state evaluation, LNs were collected 24 h after injections were given, and for inflammation conditions, injections were given at immunization. Mice were anesthetized by isoflurane inhalation (IsoFlo, Abbott) for all injection-based experiments, or anesthetized with a ketamine (100 mg per kg body weight)/xylazine (10 mg per kg body weight)/acepromazine (3 mg per kg body weight) mixture for in vivo imaging experiments.

### Histology and imaging

LNs were fixed in 4% paraformaldehyde (Electron Microscopy Sciences) in PB (0.1 M, pH 7.4) at 4 °C overnight. For cryosections, tissues were additionally embedded for 24 h in a solution of 30% glucose in PB (0.1 M, pH 7.4) before embedding and freezing in Tissue-Tek optimum cutting temperature (OCT) compound (Sakura). Cryostat sections (10–12 μm) were collected on Superfrost/Plus glass slides (Thermo Fisher Scientific). Alternatively, fixed tissues were embedded in 4% low melting temperature agarose (Invitrogen) after fixation and 100–400 µm sections were cut using a vibratome (VT1200S, Leica Microsystems).

Cryostat sections were air-dried for 2 h at room temperature (RT) and washed in PBS. Sections were blocked in SEABLOCK blocking buffer (Thermo Fisher Scientific) or in 5% bovine serum albumin (BSA; Thermo Fisher Scientific) in PBS for 1 h, followed by incubation of primary antibody solution diluted in 1% BSA/PBS for 1.5 h at RT, three washing steps in PBS and subsequent incubation of secondary antibody solution diluted in 1% BSA/PBS for 30 min at RT. Finally, sections were washed three times in PBS, air-dried and mounted using Fluoromount-G with DAPI (Thermo Fisher Scientific). Vibratome sections were blocked in 5% BSA/0.3% Triton-X/PBS for 2 h at RT under agitation followed by primary antibody incubation in 1% BSA/0.3% Triton-X/PBS overnight at 4 °C under agitation or for 2 days at RT in some cases. The following day sections were washed three times in PBS. In case primary antibodies were not conjugated with a fluorescent dye, samples were incubated with a secondary antibody in 1% BSA/0.3% Triton-X/PBS for 4 h at RT under mild agitation and subsequently washed three times in PBS. All samples were then incubated in DAPI solution for 15 min and mounted on a glass slide using Fluoromount-G (Thermo Fisher Scientific). The following primary antibodies were used: α-CD3ε-AF488 (17A2; eBioscience), α-B220-Biotin (RA3-6B2; eBioscience), α-Collagen IV-Biotin (Abcam), α-CCL21-Biotin (R&D Systems), α-PDPN-Biotin (8.1.1; eBioscience), α-PDGFR-β (R&D Systems), α-YAP/TAZ (D24E4; Cell Signal), α-cleaved Caspase 3-AF647 (Asp175; Cell Signal), α-Ki67-APC (SolA15; eBioscience), α-ICAM-1 (YN1/1.7.4; BioXCell), α-VCAM-1 (Phe25-Glu698; R&D Systems), α-Fibroblast Marker-AF647 (ERT-R7; Santa Cruz Biotech), α-CD34-FITC (RAM34; Thermo Fisher Scientific) and α-αSMA-AF488 (1A4; Thermo Fisher Scientific). α-peripheral node addressin (PNAd; MECA-79) was derived from a concentrated hybridoma supernatant (kind gift from C. Moussion). The following secondary antibodies were used: Streptavidin-Cy3 (Sigma-Aldrich), Streptavidin-AF647 (Jackson ImmunoResearch), chicken α-goat AF488 (Invitrogen), donkey α-rat AF647 (Jackson ImmunoResearch), donkey α-rabbit AF647 (Jackson ImmunoResearch) and goat α-mouse IgM AF647 (Invitrogen). Images were acquired on a Zeiss LSM 800 inverted confocal laser scanning microscope (CLSM) with the following objectives: ×10/0.45 NA, ×20/0.8 NA, ×40/1.2 NA water and ×63/1.4 NA oil Plan-APOCHROMAT.

Thick vibratome sections were in some cases cleared using the Ce3D protocol as described previously^52^. Briefly, following antibody staining, samples were washed at RT on a shaker for 8 h in washing buffer (PBS/0.3% Triton X-100, 0.5% 1-thioglycerol), which was refreshed after 4 h. Next, samples were cleared in freshly prepared Ce3D solution for 2× 1 h, mounted in µ-dishes (Ibidi) and submerged in Ce3D solution. A cover glass was placed on top to mount cleared samples to the bottom of the well and the dishes were sealed with parafilm. Large 3D volumes (*xy*: 306 × 306 µm, *z*: 50–300 µm) were acquired from Ce3D-cleared thick vibratome sections using a spinning-disk microscope (Dragonfly, Andor) with an Apochromat LWD λS ×40/1.15 water 0.60-mm WD objective.

### 3D light-sheet fluorescence microscopy sample preparation and imaging

For compartment volume analysis, intact LNs were fixed in 4% paraformaldehyde in PBS overnight at 4 °C, washed and cleaned under a stereomicroscope. LNs were subsequently blocked for 4 h at 37 °C and stained with 1 µg ml^−1^ rabbit polyclonal anti-LYVE-1 (RELIATech) for 3 days at 37 °C. After two wash steps, LNs were stained with 2 µg ml^−1^ anti-B220-AF488 (RA3-6N2; BioLegend) and 2 µg ml^−1^anti-CD3ε-AF647 (145-2C11; BioLegend) for 5 days at 37 °C. All washes and staining procedures were performed in 2% BSA/0.1% Triton X-100 in PBS, under continuous rotation at 15 r.p.m. Stained LNs were embedded in 2% low melting point agarose (Sigma-Aldrich), dehydrated in methanol and optically cleared using Murray’s clear (2:1 mix of benzyl benzoate and benzyl alcohol).

For TRC cluster analysis, terminally anesthetized Ccl19-*Cre* hem MADM-7^GT/TG^ mice (mix C57BL/6J and CD-1 background) were in vivo stained by retro-orbital injection of 40 µg mouse α-PNAd (in PBS) concentrated hybridoma supernatant labeled with Atto-647N-NHS (Atto-Tec). After 10 min, popliteal LNs were collected and fixed in 4% paraformaldehyde (Electron Microscopy Sciences) overnight at 4 °C. Samples were washed in PBS, cleaned under a stereomicroscope and cleared with the CUBIC protocol^53^. Briefly, samples were incubated in CUBIC reagent 1 for 3 days at 37 °C, which was replaced every 24 h. Samples were then washed with PBS, embedded in 2% low melting temperature agarose (Sigma-Aldrich), and sequentially dehydrated in 30% (wt/wt) sucrose (Sigma-Aldrich; 1 day at 4 °C) and 50% (wt/vol) sucrose (2 d at 4 °C). Finally, samples were incubated in CUBIC reagent 2 for 2 d at RT.

Cleared samples were imaged using a custom LSFM setup^54^. Acquired images were stitched using the Fiji Grid/Collectionstitching plugin (Preibitsch Laboratory), despeckled and, when necessary, manually registered using a custom alignment tool in MATLAB (developed by E. Papusheva).

### Lymph node compartment size analysis

Lymphatics (LYVE-1) and B cell (B220) channels were first segmented using Ilastik’s pixel classification function before being merged with the T cell (CD3ε) channel and imported into Imaris (Bitplane). Using the surface detection feature 3D volumes of the lymphatics, B follicles and the T-zone were generated. T-zone clusters in germinal centers and B cell clusters in medullary areas were excluded. To refine the volume segmentation, 3D volumes were masked and sent to Fiji. Here, small segmentation defects were manually corrected (in some cases follicle outlines, or the T-zone center where antibody penetration was suboptimal) and channels subtracted from each other to eliminate overlapping volumes (lymphatics from both B cell and T cell channel, and T cell from B cell channel). The refined segmented image was then again imported in Imaris where new 3D volumes were generated using the surface detection feature. Surface volumes were exported to Excel worksheets using the statistics tab. A custom Python script combined all data and generated volume fractions for each LN’s T-zone, follicles and lymphatics volume as a fraction of their sum.

### T-zone reticular cell cluster analysis

MADM-labeled cells were detected using a 3D approach with a spot detection algorithm (Imaris) for each channel (tdTomato and GFP) separately. Chromatic aberrations and the sequential nature of the image acquisition led to a channel misalignment, which was corrected for using the following method: the spot coordinates were exported from Imaris and treated as a point cloud for each channel. These point clouds were then registered onto each other using the Iterative closest point algorithm which corrected the shift and the rotation of the spectral channels. Cells were then sorted into color classes (green (GFP^+^), red (tdTomato^+^) or yellow (GFP^+^ tdTomato^+^) lineage). Red or green, if the spot existed solely in one of the point clouds, or yellow if there were two corresponding spots in both channels that are closer than the typical cell radius.

For the cluster analysis, the LN outline and the HEVs were segmented in Imaris using the surface detection feature. To correct errors in the cell detection, falsely detected spots from autofluorescent structures outside the LN volume were excluded from further analysis. To avoid edge effects, cells in a region 100 µm from the surface of the analyzed LNs were excluded. TRC clusters were analyzed using a custom MATLAB script utilizing a density-based spatial clustering of applications with noise (DBSCAN) algorithm^39^ in which TRCs were represented as 3D spheres with a 12-µm diameter. A TRC cluster was defined as a minimum of three TRCs of the same lineage (green or red) within a search radius of 20 µm from each TRC sphere’s surface. For visualization of cluster volumes, the convex hull of individual clusters was generated.

To generate a random distribution (simulated TRCs), TRC spheres were placed into the same volume occupied by the real cells and excluded from HEV volumes. For each time point, the average of ten distributions was used. The CF was defined as TRCs in clusters/total number of TRCs divided by simulated TRCs in clusters/total number of simulated TRCs. Cluster analysis on the processed data was performed blinded to the conditions of the experiment.

### Stromal network gap analysis

For 2D analysis, a confocal laser scanning microscope with a 40× 1.2 water objective (LSM 800, Zeiss) was used to acquire image *z*-stacks (range 10–30 µm and spaced at 1 µm) with a field of view of 240 × 240 µm and a pixel size of 0.5 µm from T- zones of FRC-mGFP mice in which TRCs are labeled, and single sections of B cell follicles of FRC-mGFP mice in which CRC stromal cells were labeled. These were subsequently segmented using Ilastik’s pixel classification feature. The result was transformed into a binary image and noise was removed using a custom Fiji script that utilized the particle detection algorithm. Binarized 3D image stacks (T-zone) and single images (B cell follicles) were then used to measure the spacing (gaps) in the network by analyzing the pore-size distribution on individual *z* sections. The pore-size distribution was obtained analogously to the pore-size analysis described in work by Acton et al.^24^. Starting with a circle size corresponding to the maximum gap of the network, circles were consecutively positioned into fitting corresponding gaps of the network. The maximum circle size was determined from a distance transform of the segmented network. Once no more circles of the maximum size could be placed into gaps of the network, the disk size was reduced by one unit and the placement of the disks of reduced sized commenced. This way, the gaps in the network were consecutively filled with circles of decreasing size until the entirety of the gap area was filled. For the T-zone, results were averaged over the image stack.

For 3D analysis, large 3D volumes (*xy*: 306 × 306 µm, *z*: 50–500 µm) were acquired from Ce3D^52^-cleared thick vibratome sections using a Apochromat LWD λS ×40/1.15 water 0.60-mm WD objective on a spinning-disk microscope (Dragonfly, Andor). Acquired 3D stacks were corrected for fluorescence intensity in the *z* axis using the ‘bleach correction (histogram matching)’ function in Fiji. Imaris was then used to generate a 3D binary image of the TRC network by utilizing a surface detection feature from the TRC network fluorescence channel. A custom MATLAB script was subsequently used to fit 3D spheres in the 3D gaps of the network analog to the 2D approach. Weighted area fractions were calculated by multiplying the area fraction for each circle (2D) or sphere (3D) with the corresponding circle or sphere diameter, respectively.

### Parallel-plate compression experiments

Explanted popliteal LNs were cleaned from adipose tissue under a stereomicroscope and placed on a glass plate within the 37 °C, RPMI 1640 (Invitrogen)-filled incubation chamber of a MicroSquisher device (CellScale). LNs were oriented to have their long axis along the field of view of the camera. Compression was performed with a glass plate glued onto either a 0.304-mm or a 0.408-mm diameter 40-GPa tungsten filament with a length of 60 mm. The glass slide on the compression probe was coated with Poly-HEMA (Sigma-Aldrich) to reduce sticking of the samples. LNs were then compressed to 75% of the initial height by lowering the upper plate down in a time span of 30 s. Lateral side views of LNs were recorded up to 20–60 min after onset of the experiment, while resistant forces were measured on the upper plate. Compression protocols, images and force acquisition were realized with the SquisherJoy software (CellScale). Length, height, contact area and curvature of LNs were manually measured before compression and at the equilibrium time points using Fiji. The recorded compression force together with the measured geometrical parameters was used to calculate volumes, Young’s modulus, effective resistance and viscosity using a generalized Kelvin model^26^. This was done as follows:

The force required to maintain a constant strain of 25% on a LN was measured over time (*F*(*t*)). The force initially peaks and then follows a relaxation curve, which is fitted by a double exponential decay curve. The simplest way to describe this bimodal dynamic is to incorporate two dashpots with constants *μ*_1_ and *μ*_2_ and two springs with *k*_1_ and *k*_2_ constants. After 20–60 min, the system reaches an equilibrium where the exerted force by the plate equals the effective resistance of the LN (*σ*). Therefore, we have:

$$\sigma = \frac{{F_{eq}/\pi R_3^2}}{{\left( {1/R1 + 1/R2} \right)}}$$

where *F*_eq_ is the equilibrium force at steady state and R1, R2 and R3 are derived from the geometry of the LN.

To obtain the elastic modulus, the stress and strain need to be acquired:

Stress (*s*) is calculated from the force at equilibrium divided by plate contact area:

$$s = F_{eq}/\pi R_3^2$$

and the strain (*ε*) from:

$$\varepsilon = 1 - \frac{{h_{eq}}}{{h_0}}$$

where *h*_0_ and *h*_eq_ correspond to the initial height and equilibrium height of the compressed LN, respectively. From here the elastic modulus (*E*) can be derived:

$$E = s/\varepsilon$$

Next, by fitting a double exponential decay to the force curve, we obtain two timescales, *τ*_1_ and *τ*_2_, where:

$$\tau _i = \mu _i/k_i,i = 1,2$$

Following up on the derivations of the equations as in work by Forgacs et al.^26^, *μ*_1_ and *μ*_2_ can be acquired readily, where *μ*_1_ corresponds to the initial fast response in the order of seconds and *μ*_2_ to the slower response in the order of minutes, of which the latter one becomes relevant for the rearrangements of the cells within LNs. Hence, we use *μ*_2_ as our viscosity.

Measurements in which the LN was damaged during preparation (lymphocytes leaking out) or moved/rolled during compression were excluded. In a few cases the viscosity could not be determined (infinitely small) and was excluded.

LN volumes were calculated from side view images at *t* *=* *0* with the following formula:

$$V = \frac{4}{3}\pi R1\left( {\frac{{h_0}}{2}} \right)^2$$

Analysis of the parallel-plate compression experiment data was performed blinded to the conditions of the experiment.

### Micropipette assay

Popliteal LN explants were cleared from fat and incubated for 10 min in 2 μg ml^−1^ ER-TR7-AF647 (Santa Cruz) in RPMI 1640 (Invitrogen) to label the capsule. LNs were subsequently placed on 3% methylcellulose-coated glass-bottom Petri dishes (MatTek) in RPMI and kept at 37 °C, while imaged on an inverted Leica SP5 microscope using a ×20, 0.7 NA objective (Leica Microsystems). The local Young’s Modulus of the capsule was measured with a glass micropipette connected to a Microfluidic Flow Control System (Fluigent, Fluiwell), with negative pressure ranging from 7 to 750 Pa, a pressure accuracy of 7 Pa and change rate of 200 Pa s^−1^. The micropipette equipment was mounted on a motorized micromanipulator (Eppendorf, Transferman Nk2). Both systems were controlled by Dikeria software, Labview (National Instruments). A fire polished micropipette with an inner diameter of 15 µm and flat end (BioMedical instruments) was used for aspiration. The chosen diameter ensured that mainly the capsule was probed and not the underlying parenchyma. While localizing the LN capsule with the micropipette, the pressure inside the micropipette was kept at 0 Pa. For measurements, a negative pressure of 750 Pa was applied, which resulted in the instantaneous aspiration of the capsule. This pressure was chosen as lower pressure regimes did not result in proper aspiration of the capsule. The tongue length of the capsule in the micropipette upon aspiration was manually measured in Fiji from acquired movies. The elasticity was subsequently calculated using Laplace’s law:

$$E = \frac{{\Delta P}}{{\left( {\frac{h}{d}} \right)^2}}$$

With Δ*P* being the pressure difference between micropipette and atmosphere, *h* the height of the measured tongue and *d* the micropipette diameter.

### Scanning electron microscope sample preparation and imaging

Terminally ketamine/xylazine/acepromazine-anesthetized mice were transcardially perfused with PB (0.1 M, pH 7.4) and subsequently fixed with 2.5% glutaraldehyde and 2% paraformaldehyde (Science Services) in PB (0.1 M, pH 7.4). LN samples were then dissected and post-fixed in the same buffer for another hour at RT. They were dehydrated in a graded ethanol series of 50%, 70%, 90%, 96% and 100% in H_2_O for a minimum of 10 min per step and subsequently kept overnight in fresh 100% ethanol at 4 °C. Once in 100% ethanol, samples were dried with a critical point dryer (EM-CPD300, Leica Microsystems), cut in half and coated with a 4-nm layer of platinum using a sputter coater (EM-ACE600, Leica Microsystems). The samples were imaged with a field emission SEM Merlin compact VP (Carl Zeiss) at 3 kV. The signal was detected by an Everhart–Thornley secondary electron detector.

### Scanning transmission electron microscopy tomography sample preparation

Alkali maceration of LNs was performed as previously described^55,56^. Briefly, popliteal LNs were isolated from 8- to 12-week-old wild-type C57BL/6 mice and directly fixed in 2.5% glutaraldehyde and 2% paraformaldehyde in PB (0.1 M, pH 7.4) for a minimum of 2 weeks at 4 °C. Samples were then macerated in aqueous 2.5 M (10% wt/vol) sodium hydroxide solution for 5 days at RT under mild agitation. Next, sections were rinsed in H_2_O under mild agitation for 1 to 2 days until samples became pale. If results were not sufficient, the maceration step was repeated.

Samples were then treated with 0.5% tannic acid (wt/vol) in PB (0.1 M, pH 7.4) twice for 1 h each with freshly prepared solutions, washed in PB and treated with aqueous 1% osmium tetroxide (wt/vol) for 30 min at 4 °C. Samples were contrast enhanced with aqueous 1% uranyl acetate (wt/vol) overnight at 4 °C and Walton’s lead aspartate for 30 min at 60 °C. Samples were then dehydrated in graded ethanol, infiltrated with anhydrous propylene oxide and embedded in hard-grade epoxy resin (Durcupan ACM, Fluca). Samples were consecutively infiltrated with a 3:1 mixture of anhydrous acetone and Durcupan for 1 h at 4 °C, 1:1 aceton/Durcupan for 1.5 h at 4 °C, 1:3 aceton/Durcupan for 2 h at 4 °C and mere Durcupan overnight at RT. Samples were transferred to BEEM capsules (Electron Microscopy Sciences), filled with freshly prepared Durcupan and cured for 48 h at 60 °C.

### Scanning transmission electron microscopy tomography imaging

Semi-thin sections were cut at 450 nm using an UC7 ultramicrotome (Leica Microsystems) and collected onto formvar-coated 200-line bar grids + 1 C/bar (Science Services, G200PB) and coated with evaporated carbon to a thickness of 8 nm. Grids were cut in half, mounted on a Half-Mesh High Tilt holder (Jeol, EM-21010/Z09291THTR) and observed under a JEM 2800 STEM (Jeol) operated at 200 kV in STEM mode. To compensate for focus, contrast and brightness and stage shift during image tilt-series recording, an automated system was used comprising the STEM Recorder V3 version 3.2.8.0 and the STEM Magica Controller version 0.9.8.1 (both System In Frontier). Images were collected at 2° intervals between ±76° of the single tilt axis. Images were captured at ×80,000–600,000 magnification, with an image size of 512 × 512 pixels (px) giving pixel sizes ranging from 6.749463 nm/px to 0.899928 nm/px.

### Conduit stretching quantification

STEM tomography images were aligned by cross-correlation and 3D structure of the area of interest computed by weighted back-projection using Composer software version 3.0 (System In Frontier). A 3D Gaussian blur filter and background subtraction (rolling ball algorithm) pre-processing step were performed on the images using Fiji. The 3D image stacks were subsequently loaded into Imaris, and fibrils of conduits were manually traced using the filament tracer feature of Imaris and exported to MATLAB format using the Object Exporter (exported from Imaris as filaments). The overall orientation and curvature of the centerline of the entire conduit was approximated by fitting a cubic spline curve with four support points, which minimized a handcrafted cost function through all fibril track data. This cost function penalizes the distance of the desired centerline to the tracks, the total curvature of the centerline and the difference in length between it and the fibril bundles and ensures that the support points are spaced evenly. In a few cases, this spline curve was corrected by hand if it was found to not adequately represent the centerline of the bundle. The alignment of the individual fibrils with respect to the centerline of the conduit was calculated as follows: the spline centerline was interpolated in a continuous fashion and the 3D orientation was calculated. Likewise, the tracks of individual fibrils were first smoothed to reduce tracing errors and the 3D orientation of each segment of the trace was calculated. The alignment angle *A* between the fibril is then given by the angle between the orientation of the segment and the orientation of the centerline at the point that is closest to the segment:

$$A = a\cos \left( {\mathrm{abs}\left( {\frac{{\mathop {v}\limits^ \to _{\mathrm{fibril}} \cdot \mathop {v}\limits^ \to _{\mathrm{centerline}}}}{{\left| {\mathop {v}\limits^ \to _{\mathrm{fibril}}} \right| \times \left| {\mathop {v}\limits^ \to _{\mathrm{centerline}}} \right|}}} \right)} \right)$$

Conduit stretching analysis was performed blinded to the conditions of the experiment.

### Ultraviolet laser cutter setup

The UV laser cutter setup is based on a previously described layout^34,57^. In brief, a passively Q-switched solid-state 355-nm UV-A laser (Powerchip, Teem Photonics) with a repetition rate of 1 kHz, pulse energy of 15 µJ, pulse length of <350 ps and peak power of 40 kW was used in conjugation with a spinning disc microscope (Andor). The system is controlled using custom-built software (LabView, National Instruments) enabling cutting in 3D. Typically, 5% of the power is used to cut tissues.

### Ultraviolet laser ablation experiments

Tension on TRCs, fLECs and capsule ECM was measured by conducting laser ablations on an inverted UV laser ablation setup with a manufacturer 40× 1.2 NA water immersion lens in homeostatic and inflamed LNs. For all experiments, 25 UV pulses at 1,000 Hz to 40 equidistant sites using a 200-ms exposure time and frame rate were used to ablate and capture tissue recoil. For TRC and fLEC ablation, we established an intravital setup where FRC-mGFP mice were anesthetized and intact inguinal LNs exposed using a skin flap surgery. The paracortical site of the LN was mounted on a custom-made stage, allowing the LN temperature to be regulated at 37 °C. For capsule ECM ablation, popliteal LNs were collected and incubated in 100 µM TAMRA in RPMI 1640 solution (both Invitrogen) for 15 min at RT and directly used for experiments. Explanted LNs were mounted at RT in a glass-bottom Petri dish (MatTek) in RPMI and prevented from moving using a 22 × 22-mm cover glass topping.

Cuts were performed in three *z* planes spaced 1 µm apart along a length of either 10 µm for TRCs or in one *z* plane along 20 µm for fLECs and capsule ECM. TRC cuts were performed in the subcapsular TRC network at IF regions, which were localized by moving online through the sample. TRCs at IF regions have bright reporter intensity and regular network morphology and are distinct from the more irregular follicle networks that contain stromal cells with both dim and spaced (CRC) and bright and dense (FDC) reporter intensity and network morphology. Recoil of TRCs and fLECs was quantified from kymographs made in Fiji, while capsule ECM recoil was quantified using PIVlab in MATLAB. In the latter case, temporal recoil velocities were measured between bandpass filtered pre-cut and consecutive post-cut frames by averaging the component of the calculated velocity in the perpendicular direction to the cut, within an area of the surrounding cut site. To demonstrate force propagation throughout the TRC network, movement vectors were created in PIVlab from the frame directly after and late after the laser cut.

### YAP/TAZ quantification

The nuclear-to-cytoplasmic ratio of stained YAP/TAZ in TRCs was measured from 3D acquisitions of peripheral LNs. In Fiji, TRCs were identified by the mGFP labeling and for each TRC the average YAP/TAZ fluorescence intensity of the nucleus (identified by DAPI) was divided by the average intensity of the adjacent cytoplasm of the cell body. In other cases, YAP/TAZ localization was qualitatively assessed to contain a higher either nuclear or cytoplasmic YAP/TAZ intensity.

### CCL21 quantification

Cryosections containing both a control and FRC^ΔTLN1^ peripheral LNs in a single section were stained for CCL21 and imaged using similar acquisition settings. The average fluorescence intensities of CCL21 within images were then measured from paracortical areas and normalized to the mean of the control samples.

### Proliferation and apoptosis measurements of T-zone reticular cells

Large 3D volumes (*xy*: 306 × 306 µm, *z*: 50–150 µm) stained for either cCasp-3 or Ki67 were acquired from Ce3D-cleared thick vibratome sections and were corrected for fluorescence intensity in *z* axis using the ‘bleach correction’ (histogram matching) function in Fiji. Imaris was then used to generate a 3D isosurface of the TRC network by utilizing a surface detection feature from the TRC network fluorescence channel. The isosurface was then used to mask the cCasp-3 and Ki67 channels so only the fluorescence signal within the TRC network remained. Positive nuclei were then manually counted from 2D slice views and normalized for per unit volume.

### Capsule thickness measurements

The thickness of capsules was measured in vibratome sections of Prox1-GFP or wild-type mice, stained for PDGFR-β and DAPI. The size of the capsule was then manually measured in Fiji from the subcapsular sinus to the surrounding adipose or muscle tissue at a minimum of three locations and were averaged per LN.

### Statistical analysis

All statistical analyses were performed in GraphPad Prism 8. *P* values < 0.05 were considered significant. No statistical methods were used to predetermine sample sizes, but our sample sizes are similar to those reported in a previous publication^24^. Normality and equal variances were formally tested to ensure the data met the assumptions of the statistical tests used.

### Software

**Table:** 

Fiji/ImageJ	Schindelin et al.^58^	https://fiji.sc/
Imaris v8.1, 9.1, 9.3, 9.8	Bitplane	https://imaris.oxinst.com/packages
Excel (v2011–2022)	Microsoft	https://products.office.com/en-us/?rtc=1
Prism v8	GraphPad	https://www.graphpad.com/scientific-software/prism/
MATLAB v2018–2021	MATLAB	https://www.mathworks.com/products/matlab.html
Ilastik, v0.5–1.1.5	Sommer et al.^59^	https://www.ilastik.org/
SquisherJoy	Cell Scale	https://www.cellscale.com/products/microtester/
Composer software	System In Frontier	https://temography.com/en/composer-en/
PIVlab	Thielicke and Stamhuis^60^	https://pivlab.blogspot.com/
Labview (2010)	National Instruments	https://www.ni.com/labview.html
ZEN blue edition v2.3	Zeiss	
Fusion v2.2	Andor	
LAS X v2.7.3.9723	Leica	

### Reporting summary

Further information on research design is available in the Nature Research Reporting Summary linked to this article.

## Online content

Any methods, additional references, Nature Research reporting summaries, source data, extended data, supplementary information, acknowledgements, peer review information; details of author contributions and competing interests; and statements of data and code availability are available at 10.1038/s41590-022-01257-4.

## Supplementary information

Supplementary Information Supplementary Tables 1–4

Reporting Summary

Supplementary Video 1 Stress relaxation experiment for which a 25% strain is applied on an explanted popliteal lymph node from a wild-type mouse (day 2 after immunization)

Supplementary Video 2 In vivo imaging of a surgically exposed inguinal lymph node in a FRC-mGFP mouse. Imaged at an IF region using a spinning disk moving from outside to the inside of the organ. Sparse lymphatic endothelial cells (LECs) of the subcapsular sinus (SCS), marginal reticular cells (MRCs) and T-zone reticular cells (TRCs) can all be observed by the FRC-mGFP labeling

Supplementary Video 3 Force propagation through the TRC network in a surgically exposed inguinal LN of a FRC-mGFP mouse. Upon an in vivo UV laser cut of a T-zone reticular cell (TRC), the local TRC network is adapting its configuration

Supplementary Video 4 Example of low (homeostasis day 0) and high tension (inflammation day 4) of the TRC network as given by the recoil speed following in vivo UV laser cutting in surgically exposed inguinal lymph nodes (LNs) of FRC-mGFP mice

Supplementary Video 5 MADM-tdTomato labeling of TRCs in 3D confocal stacks of T-zones. In homeostasis (day 0) sparse labeling of TRCs are observed, while in inflammation (day 8) large clusters of TRCs are found

## Data Availability

Source data are provided with this paper.

## Source data

Source Data Fig. 1 Statistical source data.
Source Data Fig. 2 Statistical source data.
Source Data Fig. 3 Statistical source data.
Source Data Fig. 4 Statistical source data.
Source Data Fig. 5 Statistical source data.
Source Data Fig. 6 Statistical source data.
Source Data Fig. 7 Statistical source data.
Source Data Extended Data Fig. 1 Statistical source data.
Source Data Extended Data Fig. 2 Statistical source data.
Source Data Extended Data Fig. 4 Statistical source data.
Source Data Extended Data Fig. 5 Statistical source data.
Source Data Extended Data Fig. 6 Statistical source data.

## Code availability

All analysis code are available upon request.
