# Supplementary material for: Multitier mechanics control stromal adaptations in the swelling lymph node
Source: Nat Immunol. 2022 Jul 11;23(8):1246–55. doi: 10.1038/s41590-022-01257-4 (PMC9355878; doi:10.1038/s41590-022-01257-4)
Supplement: Supplementary file 1 — Supplementary Tables 1–4 [file 41590_2022_1257_MOESM1_ESM.pdf]

nature portfolio

https://doi.org/10.1038/s41590-022-01257-4

Supplementary information

Multitier mechanics control stromal adaptations in the swelling lymph node

In the format provided by the authors and unedited

Figure	Test and Test Statistic	degrees of freedom (df) and sample size (n)	P-value	Posthoc test	Transformation
1a	Linear regression; R^2=0.8611	n=46			
1d	Kruskal-Wallis test; Chi square=22.24	df=4, n(D0/D2/D4/D8/D14)=8/11/8/9/10	P=0.0002	Dunn's. For details see table S2.	
1e	Kruskal-Wallis test; Chi square=11.68	df=4, n(D0/D2/D4/D8/D14)=8/11/7/6/10	P=0.0199	Dunn's. For details see table S2.	
1f	Kruskal-Wallis test; Chi square=9.958	df=4, n(D0/D2/D4/D8/D14)=8/11/8/9/10	P=0.0411	Dunn's. For details see table S2.	
1h_left	Two-tailed; Unpaired t test; t=6.008	df=18, n(control/anti-CD62L)=11/9	P<0.0001		
1h_right	Two-tailed; Mann-Whitney test; U=23.50	n(control/anti-CD62L)=11/9	P=0.0482		
1j_left	Two-way ANOVA; F(interaction)= 6.992, F(treatment)=80.53, F(genotype) =10.60	df=1, n(wt-PBS/wt-anti-CD62L)=13/16, n(OTII-PBS/OTII-anti-CD62L)=8/12	P(interaction)=0.0112, P(treatment)<0.0001, P(genotype)=0.0022	Tukey. For details see table S2.	Y=(Y^0.8 -1)/0.8
1j_right	Two-way ANOVA; F(interaction)= 1.843, F(treatment)=33.75, F(genotype)=8.455	df=1, n(wt-PBS/wt-anti-CD62L)=13/16, n(OTII-PBS/OTII-anti-CD62L)=8/11	P(interaction)=0.1815, P(treatment)<0.0001, P(genotype)=0.0057	Tukey. For details see table S2.	Y=Ln(Y)
2b_left	Ordinary one-way ANOVA; F=66.14	df=2,n(D0/D4/D14)=10/10/8	P<0.0001	Tukey. For details see table S2.	
2c_left	Ordinary one-way ANOVA; F=61.04	df=2,n(D0/D4/D14)=10/10/8	P<0.0001	Tukey. For details see table S2.	
2d_left	Ordinary one-way ANOVA; F=96.61	df=2,n(D0/D4/D14)=10/10/8	P<0.0001	Tukey. For details see table S2.	
2b_right	Ordinary one-way ANOVA; F=5.226	df=2,n(D0/D4/D14)=10/10/8	P=0.0127	Tukey. For details see table S2.	
2c_right	Ordinary one-way ANOVA; F=6.553	df=2,n(D0/D4/D14)=10/10/8	P=0.0052	Tukey. For details see table S2.	
2d_right	Ordinary one-way ANOVA; F=2.283	df=2,n(D0/D4/D14)=10/10/8	P=0.1228	Tukey. For details see table S2.	
2g	Ordinary one-way ANOVA; F=18.02	df=4, n(D0/D2/D4/D8/D14)=28/26/31/31/32	P<0.0001	Tukey. For details see table S2.	
3d	Kruskal-Wallis test; Chi square=177.8	df=3, n(D0/D2/D4/D14)=437/244/502/478	P<0.0001	Dunn's. For details see table S2.	
4c	Kruskal-Wallis test; Chi square=67.22	df=4, n(D0/D2/D4/D8/D14)=43/33/35/51/36	P<0.0001	Dunn's. For details see table S2.	
4f	Kruskal-Wallis test; Chi square=105.1	df=4, n(D0/D2/D4/D8/D14)=46/19/46/48/50	P<0.0001	Dunn's. For details see table S2.	
5b_left	Ordinary one-way ANOVA; F=56.10	df=2, n(D0/D4/D8)=5/5/5	P<0.0001	Tukey. For details see table S2.	
5b_right	Kruskal-Wallis test; Chi square=9.780	df=2, n(D0/D4/D8)=5/5/5	P=0.0012	Dunn's. For details see table S2.	
5e	Kruskal-Wallis test; Chi square=7.760	df=2, n(D0/D4/D8)=5/5/5	P=0.0117	Dunn's. For details see table S2.	
5g	Two-tailed; Spearman correlation; r: depicted in figure panel	n=15	see matrix in figure panel, confidence intervals see table 3		
6a_left	Two-tailed; Unpaired t test; t(D0/D4/D14)=3.015/2.390/11.41	df(D0/D4/D14)=11/29/14, n(D0-control/D0-frcΔtln1)=6/7, n(D4-control/D4-frcΔtln1)=12/19, n(D14-control/D14-frcΔtln1)=8/8	P(D0/D4/D14)=0.0118/0.236/0.0001		
6a_right	Non-linear fit. R^2(control/frcΔtln1)=0,8954/0,8904	n(control/frcΔtln1)=22/24			
6c	Two-sided; Fisher's exact test	n(D0-control/frcΔtln1)=35/84, n(D4-control/frcΔtln1)=152/134	P(D0/D4)=<0.0001/<0.0001	For details see table S4	
6e	Ordinary one-way ANOVA; F=11.65	df=5, n(D0-control/D0-frcΔtln1/D4-control/D4-frcΔtln1/D14-control/D14-frcΔtln1)=9/7/15/8/6/9	P<0.0001	Holm-Sidak. For details see table S2.	Y=Ln(Y)
6f	Two-tailed; Unpaired t test; t(D0/D4/D8)=1.094/3.222/3.138	df(D0/D4/D8)=8/8/8, n(D0-control/D0-frcΔtln1)=5/5, n(D4-control/D4-frcΔtln1)=5/5, n(D14-control/D14-frcΔtln1)=5/5	P(D0/D4/D8)=0.3056/0.0122/0.0138		
6g	Two-tailed; Unpaired t test; t(D0/D4/D8)=1.062/1.535/3.475	df(D0/D4/D8)=8/8/8, n(D0-control/D0-frcΔtln1)=5/5, n(D4-control/D4-frcΔtln1)=5/5, n(D14-control/D14-frcΔtln1)=5/5	P(D0/D4/D8)=0.3191/0.1634/0.0084		
7b	Kruskal-Wallis test; Chi square=23.66	df=4, n(D0/D2/D4/D8/D14)=11/12/20/25/22	P<0.0001	Dunn's. For details see table S2.	
7d	Ordinary one-way ANOVA; F=4.791	df=4, n(D0/D2/D4/D8/D14)=20/14/18/24/14	P=0.0016	Sidak. For details see table S2.	
7f	Kruskal-Wallis test; Chi square=24.34	df=4, n(D0/D2/D4/D8/D14)=6/6/8/7/5	P<0.0001	Dunn's. For details see table S2.	
7h	Kruskal-Wallis test; Chi square=13.65	df=4, n(D0/D2/D4/D8/D14)=8/9/9/4/6	P=0.0085	Dunn's. For details see table S2.	
7i	Kruskal-Wallis test; Chi square=24.20	df=4, n(D0/D2/D4/D8/D14)=6/6/8/7/5	P<0.0001	Dunn's. For details see table S2.	
ED 1b	Ordinary one-way ANOVA; F=69.10	df=4, n(D0/D2/D4/D8/D14)=8/11/8/9/10	P<0.0001	Holm-Sidak. For details see table S2.	
ED 1c	Non-linear fit; R^2=0.9779	n=15			
ED 1h_left	Two-tailed; Unpaired t test; t=2.693	df=18, n(control/anti-CD62L)=11/9	P=0.0149		
ED 1h_right	Two-tailed; Unpaired t test; t=0.2246	df=18, n(control/anti-CD62L)=11/9	P=0.8248		
ED 1i_left	Two-way ANOVA; F(interaction)= 3.320, F(treatment)=2.249, F(genotype)=0.03548	df=1, n(wt-PBS/wt-anti-CD62L)=13/16, n(OTII-PBS/OTII-anti-CD62L)=8/11	P(interaction)=0.0752, P(treatment)=0.1409, P(genotype)=0.8515	Tukey. For details see table S2.	Y=Ln(Y)
ED 1i_right	Two-way ANOVA; F(interaction)= 0.9930, F(treatment)=7.342, F(genotype)=2.779	df=1, n(wt-PBS/wt-anti-CD62L)=13/16, n(OTII-PBS/OTII-anti-CD62L)=8/11	P(interaction)=0.3245, P(treatment)=0.0096, P(genotype)=0.1026	Tukey. For details see table S2.	Y=Ln(Y)
ED 2g	Ordinary one-way ANOVA; F=2.360	df=2,n(D0/D4/D14)=6/6/7	P=0.1264	Dunnett. For details see table S2.	
ED 4d	Ordinary one-way ANOVA; F=11.80	df=4, n(D0/D2/D4/D8/D14)=5/5/5/5/5	P<0.0001	Dunnett. For details see table S2.	
ED 5e	Kruskal-Wallis test; Chi square=9.420	df=2, n(D0/D4/D8)=5/5/5	P=0.0024	Dunn's. For details see table S2.	
ED 5f	Kruskal-Wallis test; Chi square=8.450	df=2, n(D0/D4/D8)=5/5/5	P=0.0063	Dunn's. For details see table S2.	
ED 5g	Kruskal-Wallis test; Chi square=2.340	df=2, n(D0/D4/D8)=5/5/5	P=0.3304	Dunn's. For details see table S2.	
ED 5h	Two-tailed; Paired t test; t(D0/D4/D8)=5.966/8.543/15.17	df(D0/D4/D8)=4/4/4, n(D0-FRCs/D0-simulated/D4-FRCs/D4-simulated/D8-FRCs/D8-simulated)=5/5/5/5/5/5	P(D0/D4/D8)=0.0040/0.0010/0.0001		
ED 6a	Two-tailed; Unpaired t test; t(popliteal)=3.102, t(inguinal)=2.240	df(popliteal control/frcΔtln1)=6/6, df(inguinal control/frcΔtln1)=7/6	P(popliteal)=0.0112, P(inguinal)=0.0467		
ED 6b	Two-tailed; Mann-Whitney test; U=0	n(control/frcΔtln1)=5/5	P=0.0079		

Table1. Statistical details

**Figure 1d**	**Dunn's multiple comparisons test**	**Summary**	**Adjusted P Value**
	D0 vs. D2	**	0.0054
	D0 vs. D4	*	0.0477
	D0 vs. D8	**	0.0066
	D0 vs. D14	****	<0.0001
**Figure 1e**	**Dunn's multiple comparisons test**	**Summary**	**Adjusted P Value**
	D0 vs. D2	ns	>0.9999
	D0 vs. D4	ns	0.1888
	D0 vs. D8	ns	0.2508
	D0 vs. D14	*	0.0103
**Figure 1f**	**Dunn's multiple comparisons test**	**Summary**	**Adjusted P Value**
	D0 vs. D2	*	0.0453
	D0 vs. D4	ns	0.2951
	D0 vs. D8	ns	0.5857
	D0 vs. D14	*	0.0143
**Figure 1i_left**	**Dunn's multiple comparisons test**	**Summary**	**Adjusted P Value**
	wt:PBS vs. wtl:anti-CD62L	****	<0.0001
	wt:PBS vs. OT2:PBS	**	0.0019
	wt:PBS vs. OT2:anti-CD62L	****	<0.0001
	wt:anti-CD62L vs. OT2:PBS	**	0.0018
	wt:anti-CD62L vs. OT2:anti-CD62L	ns	0.9649
	OT2:PBS vs. OT2:anti-CD62L	***	0.001
**Figure 1i_right**	**Tukey's multiple comparisons test**	**Summary**	**Adjusted P Value**
	wt:PBS vs. wtl:anti-CD62L	****	<0.0001
	wt:PBS vs. OT2:PBS	*	0.0341
	wt:PBS vs. OT2:anti-CD62L	****	<0.0001
	wt:anti-CD62L vs. OT2:PBS	ns	0.2047
	wt:anti-CD62L vs. OT2:anti-CD62L	ns	0.6427
	OT2:PBS vs. OT2:anti-CD62L	*	0.0319
**Figure 2b_left**	**Tukey's multiple comparisons test**	**Summary**	**Adjusted P Value**
	D0 vs. D4	****	<0.0001
	D0 vs. D14	****	<0.0001
	D4 vs. D14	****	<0.0001
**Figure 2c_left**	**Tukey's multiple comparisons test**	**Summary**	**Adjusted P Value**
	D0 vs. D4	***	0.0002
	D0 vs. D14	****	<0.0001
	D4 vs. D14	****	<0.0001
**Figure 2d_left**	**Tukey's multiple comparisons test**	**Summary**	**Adjusted P Value**
	D0 vs. D4	****	<0.0001
	D0 vs. D14	****	<0.0001
	D4 vs. D14	****	<0.0001
**Figure 2b_right**	**Tukey's multiple comparisons test**	**Summary**	**Adjusted P Value**
	D0 vs. D4	ns	0.0717
	D0 vs. D14	*	0.0133
	D4 vs. D14	ns	0.6482
**Figure 2c_right**	**Tukey's multiple comparisons test**	**Summary**	**Adjusted P Value**
	D0 vs. D4	ns	0.0585
	D0 vs. D14	*	0.0047
	D4 vs. D14	ns	0.4426
**Figure 2d_right**	**Tukey's multiple comparisons test**	**Summary**	**Adjusted P Value**
	D0 vs. D4	ns	0.4399
	D0 vs. D14	ns	0.1063
	D4 vs. D14	ns	0.6162
**Figure 2g**	**Tukey's multiple comparisons test**	**Summary**	**Adjusted P Value**
	D0 vs. D2	****	<0.0001
	D0 vs. D4	***	0.0001
	D0 vs. D8	ns	0.9835
	D0 vs. D14	ns	0.9994
	D2 vs. D4	ns	0.3085
	D2 vs. D8	****	<0.0001
	D2 vs. D14	****	<0.0001
	D4 vs. D8	***	0.0006
	D4 vs. D14	****	<0.0001
	D8 vs. D14	ns	0.9348
**Figure 3d**	**Dunn's multiple comparisons test**	**Summary**	**Adjusted P Value**
	D0 vs. D2	****	<0.0001
	D0 vs. D4	****	<0.0001
	D0 vs. D14	***	0.0004
	D2 vs. D4	ns	>0.9999
	D2 vs. D14	****	<0.0001
	D4 vs. D14	****	<0.0001
**Figure 4c**	**Dunn's multiple comparisons test**	**Summary**	**Adjusted P Value**
	D0 vs. D2	ns	0.7009
	D0 vs. D4	****	<0.0001
	D0 vs. D8	**	0.0024
	D0 vs. D14	ns	>0.9999
**Figure 4f**	**Dunn's multiple comparisons test**	**Summary**	**Adjusted P Value**
	D0 vs. D2	ns	0.7348
	D0 vs. D4	****	<0.0001
	D0 vs. D8	****	<0.0001
	D0 vs. D14	*	0.0266
**Figure 5b_left**	**Dunnett's multiple comparisons test**	**Summary**	**Adjusted P Value**
	D0 vs. D4	****	<0.0001
	D0 vs. D8	****	<0.0001
**Figure 5b_right**	**Dunn's multiple comparisons test**	**Summary**	**Adjusted P Value**
	D0 vs. D4	**	0.006
	D0 vs. D8	*	0.0392
**Figure 5e**	**Dunn's multiple comparisons test**	**Summary**	**Adjusted P Value**
	D0 vs. D4	ns	0.0954
	D0 vs. D8	*	0.0144
**Figure 6e**	**Holm-Sidak's multiple comparisons test**	**Summary**	**Adjusted P Value**
	D0 control vs. D0 FRCΔTLN1	*	0.0152
	D4 control vs. D4 FRCΔTLN1	****	<0.0001
	D14 control vs. D14 FRCΔTLN1	**	0.0049
	D0 control vs. D4 control	ns	0.1164
	D4 control vs. D14 control	ns	0.2006
	D0 FRCΔTLN1 vs. D4 FRCΔtTLN1	*	0.0167
	D4 FRCΔTLN1 vs. D14 FRCΔTLN1	ns	0.1164
**Figure 7b**	**Dunn's multiple comparisons test**	**Summary**	**Adjusted P Value**
	D0 vs. D2	**	0.0050
	D0 vs. D4	ns	>0.9999
	D0 vs. D8	ns	>0.9999
	D0 vs. D14	ns	>0.9999
**Figure 7d**	**Tukey's multiple comparisons test**	**Summary**	**Adjusted P Value**
	D0 vs. D2	*	0.0106
	D0 vs. D4	**	0.0015
	D0 vs. D8	ns	0.113
	D0 vs. D14	ns	0.4728
	D2 vs. D4	ns	0.9972
	D2 vs. D8	ns	0.7166
	D2 vs. D14	ns	0.5249
	D4 vs. D8	ns	0.4143
	D4 vs. D14	ns	0.278
	D8 vs. D14	ns	0.9874
**Figure 7f**	**Dunn's multiple comparisons test**	**Summary**	**Adjusted P Value**
	D0 vs. D2	ns	>0.9999
	D0 vs. D4	ns	>0.9999
	D0 vs. D8	**	0.005
	D0 vs. D14	***	0.0002
**Figure 7h**	**Dunn's multiple comparisons test**	**Summary**	**Adjusted P Value**
	D0 vs. D2	ns	>0.9999
	D0 vs. D4	ns	>0.9999
	D0 vs. D8	ns	>0.9999
	D0 vs. D14	**	0.0082
**Figure 7i**	**Dunn's multiple comparisons test**	**Summary**	**Adjusted P Value**
	D0 vs. D2	ns	>0.9999
	D0 vs. D4	ns	>0.9999
	D0 vs. D8	**	0.0056
	D0 vs. D14	***	0.0003
**Figure S1b**	**Holm-Sidak's multiple comparisons test**	**Summary**	**Adjusted P Value**
	D0 vs. D2	**	0.0096
	D0 vs. D4	****	<0.0001
	D0 vs. D8	****	<0.0001
	D0 vs. D14	****	<0.0001
**ED Figure 1i_left**	**Tukey's multiple comparisons test**	**Summary**	**Adjusted P Value**
	wt:PBS vs. wtl:anti-CD62L	ns	0.0522
	wt:PBS vs. OT2:PBS	ns	0.7013
	wt:PBS vs. OT2:anti-CD62L	ns	0.776
	wt:anti-CD62L vs. OT2:PBS	ns	0.6537
	wt:anti-CD62L vs. OT2:anti-CD62L	ns	0.4288
	OT2:PBS vs. OT2:anti-CD62L	ns	0.9968
**ED Figure 1i_right**	**Tukey's multiple comparisons test**	**Summary**	**Adjusted P Value**
	wt:PBS vs. wtl:anti-CD62L	*	0.0247
	wt:PBS vs. OT2:PBS	ns	0.3029
	wt:PBS vs. OT2:anti-CD62L	*	0.0137
	wt:anti-CD62L vs. OT2:PBS	ns	0.8895
	wt:anti-CD62L vs. OT2:anti-CD62L	ns	0.9562
	OT2:PBS vs. OT2:anti-CD62L	ns	0.6921
**ED Figure 2g**	**Dunnett multiple comparisons test**	**Summary**	**Adjusted P Value**
	D0 vs. D4	ns	0.627
	D0 vs. D14	ns	0.4747
	D4 vs. D14	ns	0.1088
**ED Figure 4d**	**Dunnett multiple comparisons test**	**Summary**	**Adjusted P Value**
	D0 vs. D2	**	0.0029
	D0 vs. D4	*	0.013
	D0 vs. D8	***	0.0001
	D0 vs. D14	ns	>0.9999
**ED Figure 5e**	**Dunn's multiple comparisons test**	**Summary**	**Adjusted P Value**
	D0 vs. D4	*	0.0116
	D0 vs. D8	*	0.0218
**ED Figure 5f**	**Dunn's multiple comparisons test**	**Summary**	**Adjusted P Value**
	D0 vs. D4	*	0.0143
	D0 vs. D8	*	0.0429
**ED Figure 5g**	**Dunn's multiple comparisons test**	**Summary**	**Adjusted P Value**
	D0 vs. D4	ns	0.2751
	D0 vs. D8	ns	0.5777

Table 2. Post-hoc tests

Confidence Intervals Figure 5g

	LN Volume	Cluster Factor	Labeled FRCs	Clusters	FRCs in Clusters
**LN Volume**		0,5137 to 0,9394	0,4594 to 0,9304	0,2773 to 0,8957	0,2340 to 0,8861
**Cluster Factor**	0,5137 to 0,9394		0,07231 to 0,8447	0,1047 to 0,8538	0,05726 to 0,8403
**Labeled FRCs**	0,4594 to 0,9304	0,07231 to 0,8447		0,8378 to 0,9830	0,7999 to 0,9786
**Clusters**	0,2773 to 0,8957	0,1047 to 0,8538	0,8378 to 0,9830		0,9356 to 0,9935
**FRCs in Clusters**	0,2340 to 0,8861	0,05726 to 0,8403	0,7999 to 0,9786	0,9356 to 0,9935	

Table 3. Confidence intervals

Contingency Table Figure 6c

	Day 0 Control	Day 0 FRCΔTLN1	Day 4 Control	Day 4 FRCΔTLN1
NC=>1	31	9	143	27
NC<1	4	75	9	107

Table 4. Contingency Table
