# Supplementary material for: Multitier mechanics control stromal adaptations in the swelling lymph node
Source: Nat Immunol. 2022 Jul 11;23(8):1246–55. doi: 10.1038/s41590-022-01257-4 (PMC9355878; doi:10.1038/s41590-022-01257-4)
Supplement: Supplementary file 2 — Reporting Summary [file 41590_2022_1257_MOESM2_ESM.pdf]

nature portfolio

Corresponding author(s): Frank P. Assen
Michael Sixt

Last updated by author(s): May 31, 2022

Reporting Summary

Nature Portfolio wishes to improve the reproducibility of the work that we publish. This form provides structure for consistency and transparency in reporting. For further information on Nature Portfolio policies, see our Editorial Policies and the Editorial Policy Checklist.

Statistics

For all statistical analyses, confirm that the following items are present in the figure legend, table legend, main text, or Methods section.

n/a	Confirmed	
☐	☒	The exact sample size (*n*) for each experimental group/condition, given as a discrete number and unit of measurement
☐	☒	A statement on whether measurements were taken from distinct samples or whether the same sample was measured repeatedly
☐	☒	The statistical test(s) used AND whether they are one- or two-sided<br>*Only common tests should be described solely by name; describe more complex techniques in the Methods section.*
☒	☐	A description of all covariates tested
☐	☒	A description of any assumptions or corrections, such as tests of normality and adjustment for multiple comparisons
☐	☒	A full description of the statistical parameters including central tendency (e.g. means) or other basic estimates (e.g. regression coefficient) AND variation (e.g. standard deviation) or associated estimates of uncertainty (e.g. confidence intervals)
☐	☒	For null hypothesis testing, the test statistic (e.g. *F*, *t*, *r*) with confidence intervals, effect sizes, degrees of freedom and *P* value noted<br>*Give P values as exact values whenever suitable.*
☒	☐	For Bayesian analysis, information on the choice of priors and Markov chain Monte Carlo settings
☒	☐	For hierarchical and complex designs, identification of the appropriate level for tests and full reporting of outcomes
☒	☐	Estimates of effect sizes (e.g. Cohen's *d*, Pearson's *r*), indicating how they were calculated

Our web collection on statistics for biologists contains articles on many of the points above.

Software and code

Policy information about availability of computer code

Data collection	ZEN blue dition version 2.3 (Zeiss), Fusion version 2.2 (Ander), LAS X version 2.7.3.9723 (LEICA), LabVIEW 2010 (National Instruments), Squisherjoy 2.05 (Cellscale), STEM Recorder V3 Vers. 3.2.8.0 and STEM Magica Controller Vers. 0.9.8.1 (both System In Frontier Inc.)
Data analysis	Prism v8 (Graphpad), Excel (Vers. 2011-2022), Imaris v8.10-9.8 (Bitplane), Matlab 2017a-2021a (Mathworks), Composer Software Vers. 3.0 (System in Frontier Inc.), ImageJ and FIJI (NIH). PIVlab (Thielicke and Stamhuis 2014), Ilastik v1.2.2-1.3.3

For manuscripts utilizing custom algorithms or software that are central to the research but not yet described in published literature, software must be made available to editors and reviewers. We strongly encourage code deposition in a community repository (e.g. GitHub). See the Nature Portfolio guidelines for submitting code & software for further information.

Data

Policy information about availability of data

All manuscripts must include a data availability statement. This statement should provide the following information, where applicable:

- Accession codes, unique identifiers, or web links for publicly available datasets
- A description of any restrictions on data availability
- For clinical datasets or third party data, please ensure that the statement adheres to our policy

Source data for all figures are available and listed in the inventory of supplemental Information.

Human research participants

Policy information about studies involving human research participants and Sex and Gender in Research.

Reporting on sex and gender	*Use the terms sex (biological attribute) and gender (shaped by social and cultural circumstances) carefully in order to avoid confusing both terms. Indicate if findings apply to only one sex or gender; describe whether sex and gender were considered in study design whether sex and/or gender was determined based on self-reporting or assigned and methods used. Provide in the source data disaggregated sex and gender data where this information has been collected, and consent has been obtained for sharing of individual-level data; provide overall numbers in this Reporting Summary. Please state if this information has not been collected. Report sex- and gender-based analyses where performed, justify reasons for lack of sex- and gender-based analysis.*
Population characteristics	*Describe the covariate-relevant population characteristics of the human research participants (e.g. age, genotypic information, past and current diagnosis and treatment categories). If you filled out the behavioural & social sciences study design questions and have nothing to add here, write "See above."*
Recruitment	*Describe how participants were recruited. Outline any potential self-selection bias or other biases that may be present and how these are likely to impact results.*
Ethics oversight	*Identify the organization(s) that approved the study protocol.*

Note that full information on the approval of the study protocol must also be provided in the manuscript.

Field-specific reporting

Please select the one below that is the best fit for your research. If you are not sure, read the appropriate sections before making your selection.

☒ Life sciences ☐ Behavioural & social sciences ☐ Ecological, evolutionary & environmental sciences

For a reference copy of the document with all sections, see nature.com/documents/nr-reporting-summary-flat.pdf

Life sciences study design

All studies must disclose on these points even when the disclosure is negative.

Sample size	No sample-size calculations were performed beforehand. An initial sample size used in each independent were based on pilot experiments, previous experience and comparable to those reported in previous publication. For some independent experiments data was pooled to achieve statistical power (specified in figure legends). Reproducibility between independent experiments was used as a determinant for adequate sample size. Groups of at least 3 mice per group were used in independent experiments, with exception for electron microscopy experiments for which at least 2 mice per group were used.
Data exclusions	Analysis of parallel plate compression experiments resulted in a few cases (5) where the viscosity could not be determined (infinitely small). This first occurred in pilot experiments and was made a pre-determined criteria for exclusion. These datapoints were excluded, while the other measures of these measurements (volume, elastic modulus, effective resistance) were used for analysis and were in line with other measurements.
Replication	Experiments were repeated at least twice on different days, and with sufficient animals per group and measurements per animal to demonstrate statistical significance. Experiments were all reliably reproducible.
Randomization	Animals of both sex were allocated to experimental groups (homeostasis/day of inflammation, treated/control) in a random manner.
Blinding	For analysis of TRC clustering, conduit fibril alignment and parallel plate compression experiments blinding was performed. In other experiments blinding was not deemed relevant as the data obtained was not subjective or practically unfeasible.

Reporting for specific materials, systems and methods

We require information from authors about some types of materials, experimental systems and methods used in many studies. Here, indicate whether each material, system or method listed is relevant to your study. If you are not sure if a list item applies to your research, read the appropriate section before selecting a response.

Materials & experimental systems

n/a	Involved in the study
	☒ Antibodies
☒	Eukaryotic cell lines
☒	Palaeontology and archaeology
	☒ Animals and other organisms
☒	Clinical data
☒	Dual use research of concern

Methods

n/a	Involved in the study
☒	ChIP-seq
☒	Flow cytometry
☒	MRI-based neuroimaging

Antibodies

Antibodies used

Antibodies used for staining (Reagent, Species; Clone, Conjugate, Dilution, Source, Cat. No):

α-CD3ε, Rat mAb; 17A2, AF488, 1/200, Biolegend, 100201
α-CD3ε, Armenian Hamster mAb; 145-2C11, AF647, 2ug/mL, Biolegend, 100322
α-B220, Rat mAb; RA3-6B2, Biotin, 1/200, Bioscience, 36-0452-85
α-B220, Rat mAb; RA3-6N2, AF488, 2ug/mL, Biolegend, 103225
α-Collagen IV, Polyclonal rabbit, Biotin, 1/200, Abcam, ab6581
α-CCL21, Polyclonal goat, Biotin, 1/100, R&D Systems, BAF457
α-PDPN-biotin, Syr. Hamster mAb; 8.1.1, 1/150, Bioscience, 13-5381-82
α-PDGFR-β, Polyclonal goat, Unconjugated, 1/150, R&D Systems, AF1042
α-YAP/TAZ, Rabbit mAb; D2E4, Unconjugated, 1/100, Cell Signal, 84185
α-cleaved Caspase 3 (Asp175), Rabbit mAb; D3E9, AF647, 1/150, Cell Signal, 96025
α-Ki67-APC, Rat mAb; SolA15, APC, 1/100, Bioscience, 17-5698-82
α-ICAM-1, Rat mAb; YN1/1.7.4, Unconjugated, 1/200, BioXCell, BE0020-1
α-VCAM-1 (Phe25-Glu698), Polyclonal goat, Unconjugated, 1/150, R&D Systems, AF643
α-PNAd; Hybridoma-derived mouse mAb; MECA-79, Unconjugated, Undiluted supernatant, ATCC, HB-9479
α-Fibroblast Marker, Rat mAb; ERTR7, AF647, 1/100, Santa-Cruz Biotech, sc-73355
α-CD34, Rat mAb; RAM34, FITC, 1/100, Thermofisher Scientific, 11-0341-85
α-αSMA, Mouse mAb; 1A4, AF488, 1/200, Thermofisher Scientific, 53-9760-82
α-Lyve-1, Polyclonal rabbit, Unconjugated, 1ug/mL, ReliaTech, 103-PA50AG

Streptavidin; Cy3, Streptomyces avidinii, 1/200-1/400, Sigma-Aldrich, S6402
α-mouse, goat, AF647, 1/200-1/400, Invitrogen, A21238
α-goat; AF488, chicken, 1/200-1/400, Invitrogen, A21467
α-rat; AF647, donkey, 1/200-1/400, Jackson ImmunoResearc, 712-606-150
α-rabbit; AF647, donkey, 1/200-1/400, Jackson ImmunoResearch, 711-606-152
Streptavidin; AF647, Streptomyces avidinii, 1/200-1/400, Jackson ImmunoResearch, 016-600-084

Antibodies used for blocking lymphocyte homing (Reagent, Species; Clone, Conjugate, Route and dose, Source, Cat. No):
α-CD62L, Rat mAb; MEL14, Unconjugated, i.v. 100 ug, BioXCell, BE0021

Validation

Antibodies are all from commercial vendors, and were validated by the manufacturers. Antibodies were tested in the laboratory using known positive and negative controls and/or according the manufacturer's specifications and titrated.

Animals and other research organisms

Policy information about studies involving animals; ARRIVE guidelines recommended for reporting animal research, and Sex and Gender in Research

Laboratory animals

Laboratory mice (mus musculus) from the following backgrounds were used: C57B/6J (wild-type, Ccl19-Cre mTmG, Ccl19-Cre Talin1fl/fl, Ccl19-Cre mTmG Talin1fl/fl, Prox1-GFP), CD-1 (MADM-7), and mixed C57B/6 & CD-1 (Ccl19-Cre MADM-7). Both sexes in the age of 6-20 weeks were used for experiments.

Wild animals

No wild animals were used in this study.

Reporting on sex

This information has not been collected.

Field-collected samples

No field-collected samples were used in this study.

Ethics oversight

All animal experiments are in accordance with the Austrian law for animal experiments. Permission was granted by the Austrian Federal Ministry of Science, Research and Economy (identification code: BMWFW 66.018/0010-WF/V/3b/2016 and 66.018/0027-WF/V/3b/2014). Experimental plans and treatment regimens were selected in consultation with IST Austria Ethical Committee. Mice were bred and maintained at the local animal facility in accordance with IST Austria Ethical Committee or purchased from Charles River and maintained at the local animal facility in accordance with IST Austria Ethical Committee taking into account national and European guidelines.

Note that full information on the approval of the study protocol must also be provided in the manuscript.
